# Methodology for the Development of the Allergic Rhinitis and Its Impact on Asthma (ARIA)‐EAACI 2024–2025 Guidelines: From Evidence‐to‐Decision Frameworks to Digitalised Shared Decision‐Making Algorithms

**DOI:** 10.1111/all.70100

**Published:** 2025-11-21

**Authors:** Jean Bousquet, Bernardo Sousa‐Pinto, Rafael José Vieira, Holger J. Schünemann, Torsten Zuberbier, Antonio Bognanni, Alkis Togias, Boleslaw Samolinski, Arunas Valiulis, Sian Williams, Anna Bedbrook, Wienczyslawa Czarlewski, Maria Jose Torres, Mohamed H. Shamji, Mário Morais‐Almeida, G. Walter Canonica, Leticia de las Vecillas, Mark S. Dykewicz, Cristina Jacomelli, Ludger Klimek, Lucas Leemann, Olga Lourenço, Yuliia Palamarchuk, Nikolaos G. Papadopoulos, Ana Margarida Pereira, Marine Savouré, Sanna K. Toppila‐Salmi, Maria Teresa Ventura, Juan Jose Yepes‐Nuñez, Alvaro A. Cruz, Giorgio Ciprandi, Bilun Gemicioglu, Mattia Giovannini, Brigita Gradauskiene, Tuomas Jartti, Miloš Jeseňák, Piotr Kuna, Violeta Kvedariene, Désirée E. Larenas‐Linnemann, Amir H. A. Latiff, Yousser Mohammad, Ken Ohta, Padukudru A. Mahesh, Isabella Pali‐Schöll, Oliver Pfaar, Frederico S. Regateiro, Nicolas Roche, Mikhail Sofiev, Luís Taborda‐Barata, Charlotte Suppli Ulrik, Marylin Valentin Rostan, Giovanni Viegi, Luo Zhang, Josep M. Antó, Tari Haahtela, Ivan Cherrez‐Ojeda, Juan Carlos Ivancevich, Nikolai Khaltaev, Arzu Yorgancioglu, Baharudin Abdullah, Mona Al‐Ahmad, Maryam Ali Al‐Nesf, Rita Amaral, Julijana Asllani, Karl‐C Bergmann, Jonathan A. Bernstein, Michael S. Blaiss, Fulvio Braido, Paulo Camargos, Pedro Carreiro‐Martins, Thomas Casale, Lorenzo Cecchi, Alessandro G. Fiocchi, Antonio F. M. Giuliano, George Christoff, Ieva Cirule, Jaime Correia‐de‐Sousa, Elisio M. Costa, Stefano Del Giacco, Philippe Devillier, Dejan Dokic, Elham Hossny, Tomohisa Iinuma, Carla Irani, Zhanat Ispayeva, Kaja Julge, Igor Kaidashev, Kazi S. Bennoor, Helga Kraxner, Inger Kull, Marek Kulus, Maciej Kupczyk, Andriy Kurchenko, Stefania La Grutta, Neven Miculinic, Lan Le Thi Tuyet, Michael Makris, Branislava Milenkovic, Sang Min Lee, Stephen Montefort, André Moreira, Joaquim Mullol, Rachel Nadif, Alla Nakonechna, Hugo E. Neffen, Marek Niedoszytko, Dieudonné Nyembue, Robyn O'Hehir, Ismail Ogulur, Yoshitaka Okamoto, Heidi Olze, Oscar Palomares, Petr Panzner, Vincenzo Patella, Ruby Pawankar, Constantinos Pitsios, Todor A. Popov, Francesca Puggioni, Santiago Quirce, Agné Ramonaité, Marysia Recto, Maria Susana Repka‐Ramirez, Graham Roberts, Karla Robles‐Velasco, Menachem Rottem, Marianella Salapatas, Joaquin Sastre, Nicola Scichilone, Juan Carlos Sisul, Dirceu Solé, Manuel E. Soto‐Martinez, Milan Sova, Pongsakorn Tantilipikorn, Ana Todo‐Bom, Vladyslav Tsaryk, Ioanna Tsiligianni, Marilyn Urrutia‐Pereira, Erkka Valovirta, Eric Van Ganse, Tuula Vasankari, Dana Wallace, De Yun Wang, Margitta Worm, Osman M. Yusuf, Mihaela Zidarn, Sara Gil‐Mata, Manuel Marques‐Cruz, Bassam Mahboub, Ignacio J. Ansotegui, Antonino Romano, Maria Cristina Artesani, Bruno Barreto, Sven Becker, Bianca Beghe, Jacques Bouchard, Melisande Bourgoin‐Heck, Luisa Brussino, Roland Buhl, Mario Calvo‐Gil, Victoria Cardona Dahl, José Antonio Castillo Vizuete, Denis Charpin, Niels H. Chavannes, Marta Chełmińska, Lei Cheng, Ekaterine Chkhartishvili, Herberto Jose Chong‐Neto, Deepa Choudhury, Derek Chu, Cemal Cingi, Enrico Compalati, Biljana Cvetkovski, Jane Da Silva, Gennaro D'Amato, Janet Davies, Danilo Di Bona, Maria V. Dimou, Maria Do Ceu Teixeira, Maria Doulaptsi, José Miguel Fuentes Pérez, Radoslaw Gawlik, Ozlem Goksel, Maximiliano R. Gómez, Sandra N. Gonzalez Diaz, Maia Gotua, Christos Grigoreas, Ineta Grisle, Maria Antonieta Guzman, Rachel House Tan, Michael Hyland, Despo Ierodiakonou, Aspasia Karavelia, Marta Kisiel, Mitja Kosnik, Vicky Kritikos, Carlo Lombardi, Matteo Martini, Cem Meço, Eris Mesonjesi, Florin Mihaltan, Marcin Moniuszko, Robert Naclerio, Sophia Neisinger, Michal Ordak, Giovanni Paoletti, Edgar Arturo Perdomo‐Flores, Nhan Pham‐Thi, Emmanuel Prokopakis, Daniela Rivero Yeverino, Giovanni Rolla, Jan Romantowski, Philip W. Rouadi, Maia Rukhadze, Daiju Sakurai, Laila Salameh, Faradiba Serpa Sarquis, Tanja Soklic Kosak, Michael Soyka, Olga Sozinova, Krzysztof Specjalski, Vesna Tomic‐Spiric, Martina Vachova, Marianne van Hage, Ilgim Vardaloglu Koyuncu, Pakit Vichyanond, Martin Wagenmann, Fanny Wai San Ko, Pascal Werminghaus, Vicky Paraskevi Xepapadaki, Yi‐Kui Xiang, João A. Fonseca

**Affiliations:** ^1^ Institute of Allergology, Charité – Universitätsmedizin Berlin Corporate Member of Freie Universität Berlin and Humboldt‐Universität zu Berlin Berlin Germany; ^2^ Fraunhofer Institute for Translational Medicine and Pharmacology ITMP, Immunology and Allergology Berlin Germany; ^3^ ARIA (Allergic Rhinitis and Its Impact on Asthma) Montpellier France; ^4^ MEDCIDS—Department of Community Medicine, Information and Health Decision Sciences, Faculty of Medicine University of Porto Porto Portugal; ^5^ CINTESIS@RISE—Health Research Network, Faculty of Medicine University of Porto Porto Portugal; ^6^ Department of Health Research Methods, Evidence, and Impact & Department of Medicine McMaster University Hamilton Ontario Canada; ^7^ Department of Biomedical Science Humanitas University, Pieve Emanuele Milan Italy; ^8^ Division of Allergy, Immunology, and Transplantation (DAIT) National Institute of Allergy and Infectious Diseases, NIH Bethesda, MD Maryland USA; ^9^ Department of Prevention of Environmental Hazards, Allergology and Immunology Medical University of Warsaw Warsaw Poland; ^10^ Institute of Clinical Medicine and Institute of Health Sciences Medical Faculty of Vilnius University Vilnius Lithuania; ^11^ Clinic of Asthma, Allergy, and Chronic Lung Diseases, Asthma & Allergy Department Vilnius Lithuania; ^12^ International Primary Care Respiratory Group IPCRG Edinburgh UK; ^13^ Medical Consulting Czarlewski Levallois France; ^14^ Allergy Unit, Málaga Regional University Hospital of Málaga Malaga University, ARADyAL Malaga Spain; ^15^ National Heart and Lung Institute (NHLI) Imperial College, and NIHR Imperial Biomedical Research Centre London UK; ^16^ Allergy Center CUF Descobertas Hospital Lisbon Portugal; ^17^ Asthma & Allergy Unit IRCCS Humanitas Research Hospital, Rozzano Milan Italy; ^18^ Department of Allergy Hospital La Paz Institute for Health Research (IdiPAZ) Madrid Spain; ^19^ Section of Allergy and Immunology Saint Louis University School of Medicine Saint Louis Missouri USA; ^20^ “Respiriamo Insieme” Association, Asthma & Allergy Center Padova Italy; ^21^ Department of Otolaryngology, Head & Neck Surgery Universitätsmedizin Mainz Mainz Germany; ^22^ Center for Rhinology and Allergology, Allergology & Rhinology Department Wiesbaden Germany; ^23^ Department of Political Science University of Zürich Zürich Switzerland; ^24^ RISE‐Health, Department of Medical Sciences, Faculty of Health Sciences University of Beira Interior Covilhã Portugal; ^25^ Finnish Meteorological Institute (FMI) Helsinki Finland; ^26^ Allergy Department 2nd Pediatric Clinic, National and Kapodistrian University of Athens Athens Greece; ^27^ PaCeIT—Patient Centered Innovation and Technologies, Center for Health Technology and Services Research (CINTESIS), Faculty of Medicine University of Porto Porto Portugal; ^28^ Allergy Unit Instituto and Hospital CUF Porto Portugal; ^29^ Barcelona Institute for Global Health (ISGlobal) Barcelona Spain; ^30^ Universitat Pompeu Fabra (UPF) Barcelona Spain; ^31^ CIBER Epidemiología y Salud Pública (CIBERESP) Barcelona Spain; ^32^ Department of Allergy, Skin and Allergy Hospital, Inflammation Center Helsinki University Hospital and University of Helsinki Helsinki Finland; ^33^ Department of Otorhinolaryngology University of Eastern Finland and the North Savo Wellbeing Services County Kuopio Finland; ^34^ Allergy and Clinical Immunology University of Bari Medical School Bari Italy; ^35^ Institute of Sciences of Food Production National Research Council (ISPA‐CNR) Bari Italy; ^36^ School of Medicine Universidad de los Andes Bogotá DC Colombia; ^37^ Pulmonology Service, Internal Medicine Section, Fundación Santa Fe de Bogotá University Hospital Bogotá DC Colombia; ^38^ Fundaçao ProAR and (Faculdade de Medicina da) Universidade Federal da Bahia Salvador Bahia Brazil; ^39^ Casa di Cura Villa Montallegro Allergology Department Genova Italy; ^40^ Department of Pulmonary Diseases and Institute of Pulmonology and Tuberculosis Istanbul University‐Cerrahpaşa, Cerrahpaşa Faculty of Medicine Istanbul Turkey; ^41^ Department of Health Sciences University of Florence Florence Italy; ^42^ Allergy Unit Meyer Children's Hospital IRCCS Florence Italy; ^43^ Department of Immunology and Allergology Lithuanian University of Health Sciences Kaunas Lithuania; ^44^ Department of Pediatrics and Adolescent Medicine University of Turku, and Turku University Hospital Turku Finland; ^45^ Institute of Clinical Immunology and Medical Genetics, Department of Paediatrics and Adolescent Medicine, Department of Pulmonology and Phthisiology Jessenius Faculty of Medicine Martin Slovakia; ^46^ Comenius University Bratislava Slovakia; ^47^ University Teaching Hospital Martin Martin Slovakia; ^48^ Division of Internal Medicine, Asthma and Allergy, Barlicki University Hospital Medical University of Lodz Lodz Poland; ^49^ Institute of Clinical Medicine, Clinic of Chest Diseases and Allergology Faculty of Medicine, Vilnius University Vilnius Lithuania; ^50^ Institute of Biomedical Sciences, Department of Pathology, Faculty of Medicine Vilnius University Vilnius Lithuania; ^51^ Center of Excellence in Asthma and Allergy Médica Sur Clinical Foundation and Hospital México City Mexico; ^52^ Allergy & Immunology Centre Pantai Hospital Kuala Lumpur Kuala Lumpur Malaysia; ^53^ National Center for Research in Chronic Respiratory Diseases Collaborating With WHO—EMRO Tishreen University School of Medicine Latakia Syria; ^54^ Al‐Sham Private University Damascus Syria; ^55^ Japan Antituberculosis Association (JATA) Fukujuji Hospital Tokyo Japan; ^56^ Department of Respiratory Medicine JSS Medical College, JSSAHER Mysore Karnataka India; ^57^ Departement of Biological Sciences and Pathophysiology University of Veterinary Medicine Vienna Austria; ^58^ Section of Rhinology and Allergy, Department of Otorhinolaryngology, Head and Neck Surgery University Hospital Marburg, Philipps‐Universität Marburg Marburg Germany; ^59^ Allergy and Clinical Immunology Department Hospitais da Universidade de Coimbra, Unidade Local de Saúde de Coimbra Coimbra Portugal; ^60^ Center for Innovative Biomedicine and Biotechnology (CIBB), Faculty of Medicine University of Coimbra Coimbra Portugal; ^61^ Institute of Immunology, Faculty of Medicine University of Coimbra Coimbra Portugal; ^62^ UBIAir—Clinical & Experimental Lung Centre and CICS‐UBI Health Sciences Research Centre University of Beira Interior Covilhã Portugal; ^63^ Pneumologie AP‐HP Centre Université de Paris Cité, Hôpital Cochin Paris France; ^64^ UMR 1016 Institut Cochin Paris France; ^65^ Inserm Equipe d’Epidémiologie Respiratoire Intégrative, CESP Villejuif France; ^66^ Department of Immunoallergology Cova da Beira University Hospital Centre Covilhã Portugal; ^67^ UBIAir‐Clinical & Experimental Lung Centre University of Beira Interior Covilhã Portugal; ^68^ Department of Respiratory Medicine Copenhagen University Hospital‐Hvidovre Copenhagen Denmark; ^69^ Institute of Clinical Medicine University of Copenhagen Copenhagen Denmark; ^70^ Pediatrics, Allergy & Immunology Latín American Society of Allergy, Asthma & Immunology (SLAAi) Montevideo Uruguay; ^71^ Pulmonary Environmental Epidemiology Unit CNR Institute of Clinical Physiology Pisa Italy; ^72^ Department of Otolaryngology, Head and Neck Surgery Beijing TongRen Hospital and Beijing Institute of Otolaryngology Beijing China; ^73^ ISGlobal Barcelona Institute for Global Health Barcelona Spain; ^74^ Skin and Allergy Hospital Helsinki University Hospital and University of Helsinki Helsinki Finland; ^75^ Universidad Espíritu Santo Samborondón Ecuador; ^76^ Respiralab Research Group Guayaquil Guayas Ecuador; ^77^ Servicio de Alergia e Immunologia Clinica Santa Isabel Buenos Aires Argentina; ^78^ Global NCD Platform Geneva Switzerland; ^79^ Department of Pulmonary Diseases Celal Bayar University, Faculty of Medicine Manisa Turkey; ^80^ Department of Otorhinolaryngology—Head and Neck Surgery, School of Medical Sciences Universiti Sains Malaysia Kubang Kerian Kelantan Malaysia; ^81^ Microbiology Department, College of Medicine Kuwait University Kuwait City Kuwait; ^82^ Adult Allergy and Immunology Division—Hamad Medical Corporation Doha Qatar; ^83^ Department of Cardiovascular and Respiratory Sciences Porto Health School, Polytechnic Institute of Porto Porto Portugal; ^84^ Department of Women's and Children's Health, Paediatric Research Uppsala University Uppsala Sweden; ^85^ Department of Internal Medicine University of Medicine Tirana Albania; ^86^ Division of Immunology, Allergy and Rheumatology, Department of Medicine University of Cincinnati College of Medicine Cincinnati Ohio USA; ^87^ Department of Pediatrics Medical College of Georgia at Augusta University Augusta Georgia USA; ^88^ Department of Internal Medicine (DIMI) University of Genoa Genoa Italy; ^89^ Respiratory and Allergy Clinic IRCCS ‐ Policlinico San Martino Genoa Italy; ^90^ Department of Pediatrics, Medical School Federal University of Minas Gerais Belo Horizonte Brazil; ^91^ CHRC, LA‐REAL, NOVA Medical School, NMS Universidade NOVA de Lisboa Lisboa Portugal; ^92^ Serviço de Imunoalergologia Hospital de Dona Estefânia Lisbon Portugal; ^93^ Division of Allergy/Immunology University of South Florida Tampa Florida USA; ^94^ SOS Allergology and Clinical Immunology USL Toscana Centro Prato Italy; ^95^ Allergy, Bambino Gesù Children's Hospital Istituto di Ricovero e Cura a Carattere Scientifico (IRCCS) Rome Italy; ^96^ Department of Internal Medicine ‘A. Murri’ and Unit of Geriatric Immunoallergology University of Bari Medical School Bari Italy; ^97^ Faculty of Public Health Sofia Medical University Sofia Bulgaria; ^98^ Latvian Association of Allergists University Children Hospital Riga Latvia; ^99^ Life and Health Sciences Research Institute (ICVS), School of Medicine University of Minho Braga Portugal; ^100^ CINTESIS@RISE, Biochemistry Lab, Faculty of Pharmacy and Competence Center on Active and Healthy Ageing University of Porto Porto Portugal; ^101^ Department of Medical Sciences and Public Health and Unit of Allergy and Clinical Immunology University Hospital “Duilio Casula”, University of Cagliari Cagliari Italy; ^102^ VIM Suresnes, UMR 0892, Pôle des Maladies des Voies Respiratoires, Hôpital Foch Université Paris‐Saclay Suresnes France; ^103^ Medical Faculty Skopje University Clinic of Pulmonology and Allergy Skopje Republic of Macedonia; ^104^ Pediatric Allergy, Immunology and Rheumatology Unit, Children's Hospital Ain Shams University Cairo Egypt; ^105^ Department of Otorhinolaryngology Chiba University Chiba Japan; ^106^ Department of Internal Medicine and Infectious Diseases St Joseph University, Hotel Dieu de France Hospital Beirut Lebanon; ^107^ Department of Allergology and Clinical Immunology, Kazakhstan Association of Allergology and Clinical Immunology Kazakh National Medical University Almaty Kazakhstan; ^108^ Institute of Clinical Medicine Children's Clinic, Tartu University Tartu Estonia; ^109^ Poltava State Medical University Immunology & Allergology Department Poltava Ukraine; ^110^ Department of Respiratory Medicine National Institute of Diseases of the Chest and Hospital Dhaka Bangladesh; ^111^ Department of Otorhinolaryngology, Head and Neck Surgery Semmelweis University Budapest Hungary; ^112^ Department of Clinical Science and Education Södersjukhuset, Karolinska Institutet Stockholm Sweden; ^113^ Sach's Children and Youth Hospital, Södersjukhuset Stockholm Sweden; ^114^ Department of Pediatric Respiratory Diseases and Allergology Medical University of Warsaw Warsaw Poland; ^115^ Department of Clinical and Laboratory Immunology, Allergology and Medical Genetics Bogomolets National Medical University Kyiv Ukraine; ^116^ Institute of Translational Pharmacology (IFT)‐National Research Council (CNR) Palermo Italy; ^117^ Croatian Pulmonary Society Clinical Center for Pulmonary Diseases Zagreb Croatia; ^118^ Asthma, COPD Outpatient Care Unit University Medical Center Hô‐Chi‐Minh Vietnam; ^119^ Allergy Unit “D Kalogeromitros”, 2nd Department of Dermatology and Venereology National & Kapodistrian University of Athens, “Attikon” University Hospital Athens Greece; ^120^ Clinic for Pulmonary Diseases, Clinical Center of Serbia, Faculty of Medicine University of Belgrade, Serbian Association for Asthma and COPD Belgrade Serbia; ^121^ Division of Respiratory Disease and Allergy, Department of Internal Medicine Dankook University College of Medicine Cheonan Republic of Korea; ^122^ Department of Medicine, Faculty of Medicine and Surgery University of Malta, MSD Msida Malta; ^123^ EPIUnit‐Institute of Public Health University of Porto, and Laboratory for Integrative and Translational Research in Population Health (ITR) Porto Portugal; ^124^ Serviço de Imunoalergologia Centro Hospitalar Universitário São João Porto Portugal; ^125^ Basic and Clinical Immunology Unit, Department of Pathology, Faculty of Medicine University of Porto Porto Portugal; ^126^ Rhinology Unit & Smell Clínic, Department of Otorhinolaryngology, Hospital Clínic Barcelona Universitat de Barcelona; FRCB‐IDIBAPS; CIBERES Barcelona Spain; ^127^ Université Paris‐Saclay, UVSQ, Univ. Paris‐Sud Villejuif France; ^128^ Inserm Équipe d'Epidémiologie Respiratoire Intégrative Villejuif France; ^129^ Imperial College Healthcare NHS Trust London UK; ^130^ University of Liverpool Liverpool UK; ^131^ Center of Allergy, Immunology and Respiratory Diseases Santa Fe Argentina; ^132^ Department of Allergology Medical University of Gdansk Gdansk Poland; ^133^ ENT Department University Hospital of Kinshasa Kinshasa Democratic Republic of Congo; ^134^ Allergy, Asthma and Clinical Immunology, Alfred Health, Department of Immunology, Central Clinical School Monash University Melbourne Victoria Australia; ^135^ Swiss Institute of Allergy and Asthma Research (SIAF) University of Zurich Davos Switzerland; ^136^ Chiba Rosai Hospital ENT Department Chiba Japan; ^137^ Chiba University Hospital Department of Otolaryngology, Head and Neck Surgery Chiba Japan; ^138^ Department of Otorhinolaryngology Charité – Universitätsmedizin Berlin Berlin Germany; ^139^ Department of Biochemistry and Molecular Biology, School of Chemistry Complutense University of Madrid Madrid Spain; ^140^ Department of Immunology and Allergology, Faculty of Medicine in Pilsen Charles University Prague Czech Republic; ^141^ Division of Allergy and Clinical Immunology, Department of Medicine “Santa Maria della Speranza” Hospital, Battipaglia Salerno Italy; ^142^ Agency of Health ASL Division of Allergy and Clinical Immunology, Department of Medicine Salerno Italy; ^143^ Postgraduate Programme in Allergy and Clinical Immunology University of Naples Federico II Naples Italy; ^144^ Department of Pediatrics Nippon Medical School Tokyo Japan; ^145^ Medical School University of Cyprus Nicosia Cyprus; ^146^ Clinic of Occupational Diseases University Hospital Sveti Ivan Rilski Sofia Bulgaria; ^147^ IRCCS Humanitas Research Center Personalized Medicine Asthma & Allergy, Rozzano Milan Italy; ^148^ Department of Pulmonology and Allergology Klaipeda National Hospital Klaipeda Lithuania; ^149^ Vilnius University Medical Faculty Department of Political Behaviour and Institutions Vilnius Lithuania; ^150^ Division of Adult and Pediatric Allergy and Immunology University of the Philippines ‐ Philippines General Hospital Manila Philippines; ^151^ Department of Allergy, Clinics Hospital National University San Lorenzo Paraguay; ^152^ Faculty of Medicine University of Southampton Southampton UK; ^153^ The David Hide Asthma and Allergy Centre St Mary's Hospital, Isle of Wight UK; ^154^ NIHR Southampton Biomedical Research Centre University Hospital Southampton NHS Foundation Trust Southampton UK; ^155^ LEADER Research Inc Hamilton Ontario Canada; ^156^ Division of Allergy, Asthma and Clinical Immunology Emek Medical Center Afula Israel; ^157^ Rappaport Faculty of Medicine Technion‐Israel Institute of Technology Haifa Israel; ^158^ ARIA, Asthma, Rhinitis, Immunology & Allergy Department Athens Greece; ^159^ Allergy Service, Fundacion Jimenez Diaz Autonoma University of Madrid, CIBERES‐ISCIII Madrid Spain; ^160^ PROMISE Department University of Palermo Palermo Italy; ^161^ Allergy & Asthma, Medical Director CLINICA SISUL, FACAAI, SPAAI Asuncion Paraguay; ^162^ Division of Allergy, Clinical Immunology and Rheumatology, Department of Pediatrics Federal University of São Paulo São Paulo Brazil; ^163^ Division of Respiratory Medicine, Department of Pediatrics, Hospital Nacional de Niños Universidad de Costa Rica San Jose Costa Rica; ^164^ Department of Respiratory Medicine and Tuberculosis University Hospital Brno Czech Republic; ^165^ Department of Otolaryngology, Faculty of Medicine Siriraj Hospital Mahidol University Bangkok Thailand; ^166^ Imunoalergologia, Centro Hospitalar Universitário de Coimbra, Faculty of Medicine University of Coimbra Coimbra Portugal; ^167^ International Primary Care Respiratory Group IPCRG Aberdeen UK; ^168^ Health Planning Unit, Department of Social Medicine, Faculty of Medicine University of Crete Heraklion Greece; ^169^ Department of Medicine Federal University of Pampa Uruguaiana Brazil; ^170^ Department of Lung Diseases and Clinical Immunology University of Turku Turku Finland; ^171^ Research on Healthcare Performance (RESHAPE), INSERM U1290, Pneumologie et Medicine Respiratoire, Groupement Hospitalier Nord Hôpital de la Croix Rousse Lyon France; ^172^ FiLHA Finnish Lung Health Association Helsinki Finland; ^173^ Department of Clinical Medicine, Pulmonary Diseases and Clinical Allergology University of Turku Turku Finland; ^174^ Nova Southeastern University College of Allopathic Medicine Fort Lauderdale Florida USA; ^175^ Department of Otolaryngology, Yong Loo Lin School of Medicine National University of Singapore Singapore Republic of Singapore; ^176^ Division of Allergy and Immunology, Department of Dermatology, Allergy and Venerology Charité – Universitätsmedizin Berlin Berlin Germany; ^177^ The Allergy and Asthma Institute Allergy & Asthma Department Islamabad Pakistan; ^178^ University Clinic of Respiratory and Allergic Diseases, Pulmonary & Allergy Department Golnik Slovenia; ^179^ Faculty of Medicine University of Ljubljana Ljubljana Slovenia; ^180^ Pulmonary Department Rashid Hospital, DUBAI Health Dubai UAE; ^181^ Research Institute of Medical and Health Sciences University of Sharjah Sharjah UAE; ^182^ Department of Allergy and Immunology Hospital Quironsalud Bizkaia Bilbao Spain; ^183^ Oasi Research Institute‐IRCCS Troina Italy; ^184^ BIOS S.p.A. Società Benefit Rome Italy; ^185^ Department of Allergy and Immunology Para State University Center ‐ CESUPA Belém Brazil; ^186^ Department for Otorhinolaryngology, Head and Neck Surgery University of Tübingen Tübingen Germany; ^187^ Respiratory Medicine, University of Modena & Reggio Emilia Azienda Ospedaliera‐Universitaria di Modena Modena Italy; ^188^ Laval University, Quebec City Quebec Canada; ^189^ Department of Pediatric Allergology Armand Trousseau University Hospital, Sorbonne University, AP‐HP Paris France; ^190^ French National Reference Center for Angioedema (CREAK) Saint‐Antoine University Hospital Paris France; ^191^ CRESS, Inserm, INRAE, HERA Team Paris Cité University Paris France; ^192^ Department of Medical Sciences University of Turin Turin Italy; ^193^ Allergy and Clinical Immunology Unit Mauriziano Hospital Torino Italy; ^194^ Department of Pulmonary Medicine Mainz University Hospital Mainz Germany; ^195^ Pediatrics Department, Faculty of Medicine Universidad Austral de Chile Valdivia Chile; ^196^ Allergy Section, Department of Internal Medicine Hospital Vall D'hebron Barcelona Spain; ^197^ ARADyAL Research Network Barcelona Spain; ^198^ Pneumology Department Hospital Universitari Dexeus Barcelona Spain; ^199^ CIBER of Respiratory Diseases Group of Rhinitis, Rhinosinusitis and Nasal Polyps, Area of Asthma Barcelona Spain; ^200^ Clinique des Bronches, Allergie et Sommeil Hôpital Nord Marseille France; ^201^ National eHealth Living Lab, Department of Public Health and Primary Care Leiden University Medical Center Leiden the Netherlands; ^202^ Department of Allergology Medical University of Gdańsk Gdańsk Poland; ^203^ Department of Allergology and Department of Otorhinolaryngology, the First Affiliated Hospital Nanjing Medical University Nanjing China; ^204^ Allergist David Tvildiani Medical University Tbilisi Georgia; ^205^ Associate Professor of Pediatrics, Division of Allergy and Immunology Federal University of Parana Curitiba Brazil; ^206^ Paediatric Allergy Clinic, Department of Dermatology Amersham Hospital ‐ NHS Hospital Trust Amersham UK; ^207^ Department of Health Research Methods, Evidence & Impact McMaster University Hamilton Ontario Canada; ^208^ Evidence in Allergy Group McMaster University and the Research Institute of St. Joe's Hamilton Hamilton Ontario Canada; ^209^ Medical Faculty, ENT Department Eskisehir Osmangazi University Eskisehir Turkey; ^210^ Scientific & Medical Department Lofarma S.p.A Milan Italy; ^211^ Woolcock Institute of Medical Research Sydney Australia; ^212^ Allergy Service University Hospital Professor Polydoro Ernani de São Thiago (HU‐UFSC/EBSERH) Florianópolis Brazil; ^213^ Department of Internal Medicine Federal University of Santa Catarina (UFSC) Florianópolis Brazil; ^214^ Division of Respiratory and Allergic Diseases, High Specialty Hospital 'a Cardarelli', and Respiratory Allergy School of Specialization in Respiratory Diseases, Federico II University of Naples Naples Italy; ^215^ Centre for Immunology and Infection Control, School of Biomedical Sciences, Faculty of Health Queensland University of Technology Brisbane Australia; ^216^ Office of Research Metro North Hospital and Health Service Brisbane Australia; ^217^ Department of Medical and Surgical Sciences, School of Allergology and Clinical Immunology University of Foggia Foggia Italy; ^218^ Dr Agostinho Neto University Hospital Epidemiology Department Praia Cabo Verde; ^219^ Immunology, Cabo Verde University Faculty of Medicine Praia Cape Verde; ^220^ Department of Otorhinolaryngology, Head and Neck Surgery University of Crete, School of Medicine, Heraklion Crete Greece; ^221^ Allergist Private Practice Mexico City Mexico; ^222^ Department of Internal Diseases, Allergology and Clinical Immunology Medical University of Silesia in Katowice Katowice Poland; ^223^ Department of Pulmonary Medicine, Division of Immunology, Allergy and Asthma, Laboratory of Occupational and Environmental Respiratory Diseases, Faculty of Medicine Ege University, EgeSAM (Ege University Translational Pulmonary Research, Center), Bornova Türkiye; ^224^ Faculty of Health Sciences Catholic University of Salta Salta Argentina; ^225^ Allergy and Clinical Immunology Centro Regional Hospital Universitario, Universidad Autónoma de Nuevo Leon Monterrey Mexico; ^226^ Center of Allergy and Immunology, and Georgian Academy of Allergy, Asthma and Clinical Immunology Tbilisi Georgia; ^227^ Department of Allergy and Clinical Immunology Air Force General Hospital Athens Greece; ^228^ Riga East University Hospital Riga Latvia; ^229^ Immunology and Allergy Division Clinical Hospital, University of Chile Santiago Chile; ^230^ Quality Use of Research Medicines Group, Woolcock Institute of Medical Research Macquarie Park NSW Australia; ^231^ Macquarie University Macquarie Park NSW Australia; ^232^ Faculty of Health University of Plymouth Plymouth UK; ^233^ Department of Primary Care and Population Health University of Nicosia Medical School Nicosia Cyprus; ^234^ Otolaryngology Department General Hospital of Kalamata Kalamata Greece; ^235^ Department of Medical Sciences, Occupational and Environmental Medicine Uppsala University Uppsala Sweden; ^236^ Clinical Management Group Woolcock Institute of Medical Research Sydney Australia; ^237^ Sydney Pharmacy School, Faculty of Medicine and Health University of Sydney Sydney New South Wales Australia; ^238^ Departmental Unit of Allergology Clinical Immunology & Pneumology, Istituto Ospedaliero Fondazione Poliambulanza Brescia Italy; ^239^ Allergy Unit, Department of Internal Medicine University Hospital AOU delle Marche Ancona Italy; ^240^ Department of Clinical and Molecular Sciences Marche Polytechnic University Ancona Italy; ^241^ Department of Otorhinolaryngology, Head and Neck Surgery Ankara University Medical School Ankara Turkey; ^242^ Department of Otorhinolaryngology Head and Neck Surgery Salzburg Paracelsus Medical University Salzburg Austria; ^243^ Department of Otolaryngology, Head and Neck Surgery Weill Cornell Medical College, Cornell University New York NY USA; ^244^ Allergy and Clinical Immunology Department University Hospital Center “Mother Teresa” Tirana Albania; ^245^ UMF‐University of Medicine and Pharmacy ‘Carol Davila’, Pneumology Department National Institute of Pneumology ‘Marius Nasta’ Bucharest Romania; ^246^ Department of Allergology and Internal Medicine Medical University of Bialystok Byalistok Poland; ^247^ Department of Otolaryngology‐Head and Neck Surgery Johns Hopkins University Baltimore Maryland USA; ^248^ Department of Pharmacotherapy and Pharmaceutical Care, Faculty of Pharmacy Medical University of Warsaw Warsaw Poland; ^249^ Otorhinolaryngology and Neck Surgery Unit San Juan de Dios National Hospital San Miguel El Salvador; ^250^ Ecole Polytechnique de Palaiseau Palaiseau France; ^251^ IRBA (Institut de Recherche Bio‐Médicale Des Armées) Brétigny sur Orge France; ^252^ Université Paris Cité Paris France; ^253^ Servicio de Alergia e Inmunología clínica Hospital Universitario de Puebla Puebla México; ^254^ Department of Otolaryngology‐Head and Neck Surgery, Eye and Ear University Hospital Beirut Lebanon; ^255^ Department of Otorhinolaryngology‐Head and Neck Surgery Dar Al Shifa Hospital Salmiya Kuwait; ^256^ Center Allergy & Immunology and Geomedi Teaching University Faculty of Medicine Tbilisi Georgia; ^257^ Department of Otorhinolaryngology, Head and Neck Surgery, Interdisciplinary Graduate School of Medicine University of Yamanashi Yamanashi Japan; ^258^ Mohammed Bin Rashid University (MBRU) Dubai Health Dubai UAE; ^259^ Asthma Reference Center—School of Medicine of Santa Casa de Misericórdia of Vitória Espírito Santo Brazil; ^260^ ORL SOKLIC KOSAK Ljubljana Slovenia; ^261^ Otolaryngology‐HNS University of Zurich, University Hospital of Zurich Zurich Switzerland; ^262^ Head of Laboratory of Atmospheric Processes and Aerobiology University of Latvia Riga Latvia; ^263^ Clinic of Allergology and Immunology University Clinical Center of Serbia Belgrade Serbia; ^264^ Faculty of Medicine University of Belgrade Belgrade Serbia; ^265^ Department of Immunology and Allergology University Hospital and Faculty of Medicine in Pilsen Czech Republic; ^266^ Charles University Prague Czech Republic; ^267^ Department of Medicine Solna, Division of Immunology and Respiratory Medicine Karolinska Institutet Stockholm Sweden; ^268^ Department of Clinical Immunology and Transfusion Medicine Karolinska University Hospital Stockholm Sweden; ^269^ Center for Molecular Medicine Karolinska University Hospital Stockholm Sweden; ^270^ Department of Chest Diseases Istanbul University‐Cerrahpaşa, Cerrahpaşa Faculty of Medicine Istanbul Turkey; ^271^ Division of Allergy and Immunology, Department of Pediatrics Siriraj Hospital, Mahidol University Faculty of Medicine Bangkok Thailand; ^272^ Samitivej Allergy Institute Bangkok Thailand; ^273^ Department of Otorhinolaryngology Heinrich Heine University Düsseldorf, Medical Faculty and University Hospital Düsseldorf Düsseldorf Germany; ^274^ Department of Medicine and Therapeutics The Chinese University of Hong Kong Hong Kong China; ^275^ ENT and Allergology Düsseldorf Germany; ^276^ Shanghai Skin Disease Hospital Tongji University School of Medicine Shanghai China

**Keywords:** allergic rhinitis, asthma, ENT (rhinitis, sinusitis, nasal polyps…), guidelines

## Abstract

The Allergic Rhinitis and its Impact on Asthma (ARIA) guidelines produced their first edition in 1999, with subsequent revisions in 2008, 2010, 2016 and 2019. A new iteration of ARIA—ARIA 2024–2025—in collaboration with EAACI is currently being developed, focusing on the management of allergic rhinitis. ARIA 2024–2025 follows the GRADE framework and is endorsed by the European Academy of Allergy and Clinical Immunology (EAACI). A set of approaches has been used to develop guideline questions, including surveying key opinion leaders and using artificial intelligence (AI)‐based tools to analyse web searches on allergic rhinitis and to generate questions. Each prioritised guideline question is assessed through an Evidence‐to‐Decision (EtD) framework. EtDs support the systematic and transparent formulation of recommendations, comprising 12 criteria for which the best available evidence should be sought. In the context of ARIA‐EAACI 2024–2025, such evidence is derived not only from randomised controlled trials but also—among others—from patient‐generated data sources that better reflect the affected individuals' perspectives. Moreover, ARIA‐EAACI 2024–2025 incorporates evidence on planetary health. Developed guideline recommendations will support the creation of digitalised decision algorithms and care pathways. This paper describes the methodology used to develop the person‐centred, digitally enabled and AI‐assisted ARIA‐EAACI 2024–2025. Among others, it describes (i) the development and prioritisation of guideline questions, (ii) sources of evidence for EtDs and (iii) the development of digitalised decision algorithms and care pathways.

## Graphical abstract

The Allergic Rhinitis and its Impact on Asthma (ARIA) group is working on the 2024–2025 update of its guidelines for the management of allergic rhinitis (ARIA 2024–2025). The ARIA 2024–2025‐EAACI guidelines will follow GRADE methodology and will incorporate evidence from different sources, including systematic reviews and meta‐analyses, mHealth data and pharmacovigilance data, among others. The ARIA‐EAACI 2024–2025 guidelines will be innovative by (i) being supported by artificial intelligence on aspects such as question generation, (ii) resulting in the production of digital algorithms for the management of allergic rhinitis and (iii) considering planetary health. AE, adverse events; AI, artificial intelligence; ARIA, Allergic Rhinitis and its Impact on Asthma; ENT, otolaryngologist; GRADE, grading of recommendations, assessment, development and evaluation; GT, Google Trends; KOL, key opinion leaders.

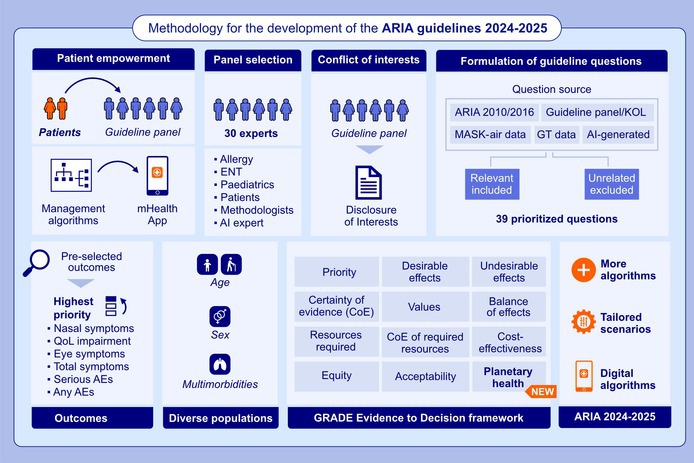

## 1 Introduction

Allergic rhinitis (AR) is one of the most common chronic conditions globally, often co‐occurring with asthma and conjunctivitis [1, 2, 3]. It impairs quality of life anaffects social life, school and work productivity, and is associated with substantial economic costs [1, 4]. AR is an excellent candidate for testing innovation in guidelines: the vast inter‐individual differences in exposures and in personal beliefs, preferences and values require a person‐centred approach. In addition, AR is often self‐managed, with limited contact with health services.

**FIGURE 1:**
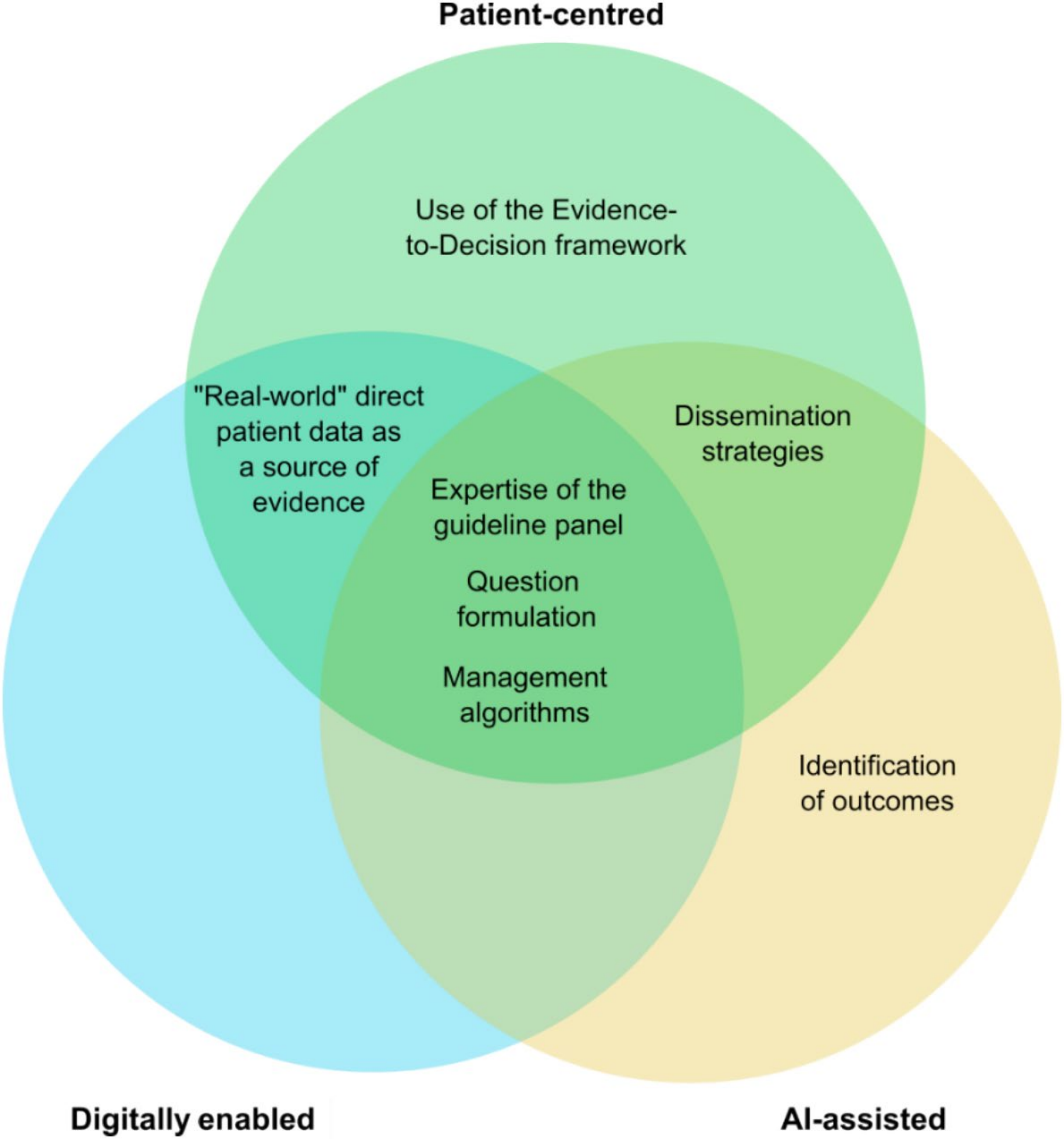
Graphical representation of the tasks of the Allergic Rhinitis and its Impact on Asthma (ARIA‐EAACI) 2024–2025 guidelines and their classification as patient‐centred, digitally enabled and artificial intelligence (AI)‐assisted.

The Allergic Rhinitis and its Impact on Asthma (ARIA) initiative first proposed guidelines for AR and asthma multimorbidity in 1999 [5]. ARIA has evolved from the first multimorbidity guideline in respiratory diseases using the Shekelle evidence‐based model [5] to the GRADE (Grading of Recommendations, Assessment, Development and Evaluation) framework for evidence‐based guidelines [6, 7]. In fact, ARIA 2016 was used as the clinical scenario on how to interpret and use a guideline in a review published in the Journal of the American Medical Association [8]. ARIA 2010 and 2016 devised a medication algorithm [6, 7]. However, digitalisation was not implemented. In 2019, next‐generation guidelines using real‐life MASK‐air data and allergen exposure chamber studies proposed digitally enabled person‐centred care for the first time [9]. The next iteration, ARIA‐EAACI 2024–2025, is endorsed by the European Academy of Allergy and Clinical Immunology and will result in a person‐centred, digitally enabled, artificial intelligence (AI)‐assisted guideline [10] (Figure 1).

ARIA‐EAACI 2024–2025 is formulating recommendations based on the use of the GRADE Evidence‐to‐Decision (EtD) framework. The EtD supports the systematic and transparent formulation of recommendations for each prioritised guideline question [11, 12]. It comprises 12 criteria for which the best available evidence should be sought. In ARIA‐EAACI 2024–2025, such evidence is not only based on data from randomised controlled trials (RCTs) but also from other sources, including studies using data directly provided by patients. In fact, although RCTs are the gold standard for assessing the efficacy of medical interventions, they present several limitations. In this context, in AR, well‐designed pragmatic trials and observational studies using direct patient data can be a source of complementary evidence (Bousquet, submitted), providing insights into the reality of day‐to‐day clinical practice.

In addition to being informed by multiple sources of evidence, ARIA‐EAACI 2024–2025 will be innovative in its final result (Table 1). In addition to recommendations, ARIA‐EAACI 2024–2025 will result in a set of personalised and digitalised algorithms that can be embedded in an app. For example, the MASK‐air mHealth app is a Class IIa medical device, in which algorithms can be tested and implemented into the clinical workflow of allergists [13, 15]. Henceforth, recommendations will be included in digitalised management algorithms considering multiple clinical scenarios. Algorithm digitalisation has several advantages, including patient empowerment and flexibility for the implementation of updates in the perspective of a living ARIA guideline. Digital divide (e.g., age‐related) aspects do however need to be considered.

**TABLE 1:** Summary of innovative approaches followed in the development of the ARIA‐EAACI 2024–2025 guidelines.

Task	“Classical” approaches followed in the ARIA‐EAACI 2024–2025 guidelines	Innovative approaches followed in the ARIA‐EAACI 2024–2025 guidelines
Formulation of guideline questions	– Identification of previous guideline questions; – Formulation of guideline questions by experts.	– Identification of questions based on direct patient‐reported data (MASK‐air app); – AI‐assisted identification of questions posed by Internet users in online searches; – Direct generation of questions by AI tools.
Identification of outcomes	– Identification of outcomes by experts.	– Identification of outcomes by AI tools.
Completing the evidence‐to‐decision frameworks		
Desirable and undesirable effects	– Use of evidence from systematic reviews (of RCTs).	– Use of pharmacovigilance data.
Values	– Use of evidence from systematic reviews of values and preferences.	– Assessment of utilities based on direct patient data (MASK‐air app) [study included in the systematic review of values and preferences].
Resources used and cost‐effectiveness	– Use of data from the scientific literature and health technology assessment reports.	– Assessment of indirect costs based on direct patient data (MASK‐air app) – Survey of ARIA experts on the costs of medications.
Equity		– Survey of ARIA experts on the availability of medication; – Systematic consideration of medications in the World Health Organisation List of Essential Medicines.
Acceptability	– Use of data from the scientific literature (e.g., for onset of action).	– Use of direct patient data (MASK‐air app) to assess adherence, frequency of co‐medication and treatment satisfaction with different medications.
Planetary Health	*(planetary health not usually considered in guidelines)*	– Consideration of planetary health.
Considering multimorbidity		– Explicit consideration of multimorbidity in subgroup considerations.
Building treatment algorithms	– Already proposed in the former ARIA iterations [13, 14]	– Building and implementation of digitalised algorithms in an AI‐assisted process involving a multidisciplinary team.

Abbreviations: AI, artificial intelligence; RCT, randomised controlled trial.

In this paper, we aim to present the methodology for the development of ARIA 2024–2025 and to provide a rationale for the creation of digitalised guideline algorithms.

## 2 ARIA‐EAACI 2024–2025: Patient‐Centred, Digitally Enabled, AI‐Assisted Guidelines

### 2.1 Patient Empowerment

ARIA‐EAACI 2024–2025 is being devised throughout the different stages on a person‐centred basis (Figure 1). First, AR patients have been included in the guideline panel. Second, to ensure that guideline questions are person‐centred, questions were formulated pertaining to (i) findings and hypotheses from studies based on data directly provided by patients in MASK‐air and (ii) AI‐supported approaches that analysed popular online search queries on AR posed by internet users [16].

Third, we are using the EtD framework to develop our recommendations—the EtDs imply that the formulation of recommendations is not solely based on the desirable and undesirable effects of the interventions, but also on aspects such as patients' values and the acceptability of interventions by the different interest holders (considering aspects such as patients' satisfaction with medications or the speed of onset of action of treatments) [11, 12].

Fourth, patient behaviours are being considered to support the formulation of recommendations. As an example, we are considering steroid phobia for recommendations related to intranasal corticosteroids (INCS) [17]. In addition, we are considering that most patients do not use medication regularly, but rather on an as‐needed basis. This has been observed in studies using MASK‐air direct patient data [18], and the few RCTs comparing as‐needed versus regular treatment for AR did not find any major differences [19, 20, 21, 22, 23, 24, 25, 26].

Finally, the ARIA‐EAACI 2024–2025 guidelines will result in the development of management algorithms that will be implemented in an mHealth app that can be accessed by patients.

### 2.2 Digital Enablement

Digital enablement is a cornerstone of ARIA‐EAACI 2024–2025: (i) findings and hypotheses from studies using mHealth data enabled the formulation of guideline questions, (ii) mHealth direct patient data are used as a source of evidence and (iii) management algorithms will be digitalised into an mHealth app (Figure 1). A market research study has analysed available mHealth apps for AR and has concluded that MASK‐air is available in the largest number of countries and has the highest number of scientific publications [27]. Moreover, MASK‐air is one of the few apps reporting asthma multimorbidity. Therefore, MASK‐air was selected as the app from which data are retrieved to provide evidence for guideline development and to implement digitalised algorithms.

MASK‐air is available in 30 countries. It has been used by more than 40,000 patients, who have reported over 700,000 days of use [15]. MASK‐air is a patient‐centred Medical Device regulation Class IIa. It is a Good Practice of the Directorate General Health and Food Safety of the European Commission [28] as well as an OECD (Organisation of Economic Cooperation and Development) Best Practice for Integrating Care to Prevent and Manage Chronic Diseases [13].

MASK‐air comprises a daily monitoring questionnaire assessing the impact of rhinitis and asthma symptoms by means of validated visual analogue scales (VASs) [29, 30]. In addition, users are asked (i) to enter their daily medications via a regularly updated scroll list that contains country‐specific prescribed and over‐the‐counter medications and (ii) to provide feedback on their satisfaction with their medication. MASK‐air also includes additional questionnaires, namely the Control of Allergic Rhinitis and Asthma Test (CARAT) [31], the Work Productivity and Activity Impairment Questionnaire: Allergy Specific (WPAI‐AS) [32, 33], and EQ‐5D [34, 35]. Model predictions of pollen concentrations and air pollution obtained from the Finnish Meteorological Institute (https://silam.fmi.fi) [36, 37] and, for some species, from Copernicus Atmosphere Monitoring Service (http://atmospere.copernicus.eu) are available daily within a 10 km radius in Europe in geolocalised patients. Table S1 presents some of the relevant findings of MASK‐air studies for the development of ARIA‐EAACI 2024–2025.

### 2.3 Use of AI

In the context of ARIA‐EAACI 2024–2025, AI helped in the development of guideline questions and the identification of outcomes (Figure 1). In particular, a large language model‐based chatbot was prompted to (i) classify online search queries into those conveying questions [16], (ii) suggest guideline questions [16] and (iii) suggest potentially relevant outcomes. In addition, AI was used to support the systematic review on patients' values and preferences in AR [38]. In ARIA‐EAACI 2024–2025, we plan to use AI to support (i) the writing of plain language summaries and (ii) the development of the digitalised algorithms.

## 3 Panel Selection

ARIA group members involved in ARIA‐EAACI 2024–2025 include (i) the steering committee, (ii) the guideline panel, (iii) the ARIA review group and (iv) the ARIA Junior Members (Table S2).

The ARIA guideline panel includes 30 experts and is responsible for (i) suggesting and prioritising guideline questions and outcomes, (ii) participating in group meetings, (iii) providing input when discussing the available evidence for each guideline question, (iv) reviewing evidence summaries, (v) making judgements for each EtD criterion, (vi) drafting guideline recommendations, (vii) reviewing and writing the final report and (viii) supporting guideline dissemination.

The guideline panel was set in such a way as to ensure (i) the inclusion of experts with different profiles (physicians specialised in allergy, otorhinolaryngology, paediatrics and primary care, pharmacists, patients, methodologists and AI experts), (ii) representativeness in terms of gender, age and country and (iii) that at least 50% of the members did not have a conflict of interest (CoI). All members were required to complete the “Guideline Group or Panel Member Certification Course” (now termed “Certified Guideline Panel Member” course) of the International Guideline Training and Certification Programme INGUIDE.

To increase the diversity of the received inputs, we invited an ARIA review group (including members from low‐ and middle‐income countries) and ARIA Junior Members to participate in certain guideline tasks. Tasks for these groups include (i) providing input on non‐prioritised questions, (ii) reviewing formulated recommendations and (iii) participating in the development of the management algorithms.

## 4 Development and Prioritisation of the Guideline Questions

**FIGURE 2:**
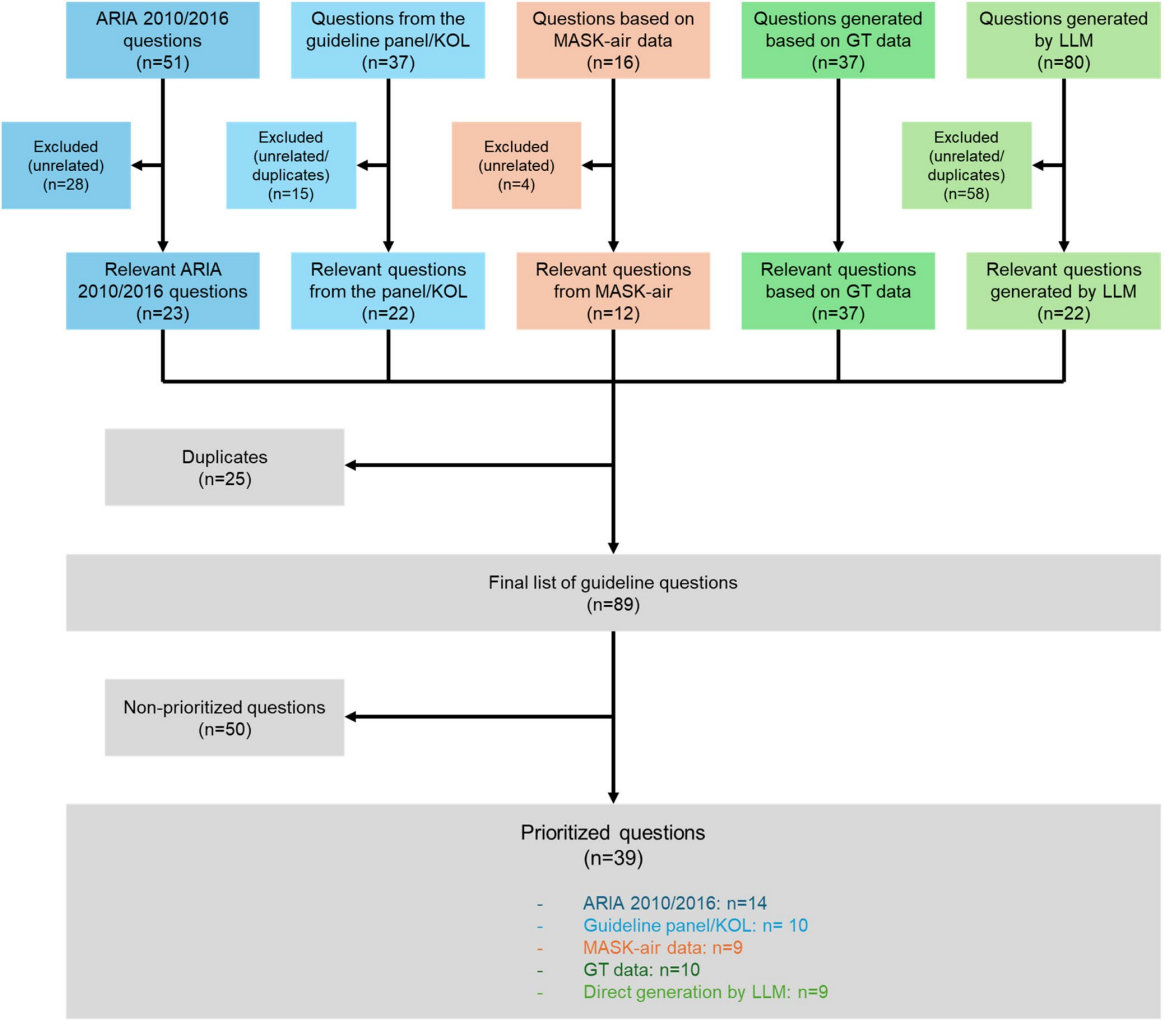
Flow diagram for the formulation of guideline questions. GT, Google Trends; KOL, key opinion leaders.

In ARIA‐EAACI 2024–2025, several different approaches were used for the development of guideline questions (Figure 2; Table 2), including person‐centred approaches. The developed questions were then prioritised to select those for which recommendations would be drafted.

**TABLE 2:** Full list of guideline questions voted for prioritisation and methods used for their generation.

Guideline question	Method for question generation					Prioritised
	ARIA 2010–2016	Guideline panel/KOL	MASK‐air data	Generation based on web search queries	Direct generation by LLM	
Should continuous long‐term treatment vs. as‐needed treatment be used in patients with allergic rhinitis?			×	×		×
Should co‐medication vs. medication updosing be used in patients with allergic rhinitis that is poorly controlled despite pharmacologic treatment?			×			×
Should pharmacologic treatment adjustment according to seasonality in pollen or other allergen exposure vs. no adjustment be used in patients with allergic rhinitis?			×			×
Should pharmacologic treatment adjustment according to air pollution levels vs. no adjustment be used in patients with allergic rhinitis?			×			×
Should intranasal H1 antihistamines vs. no treatment be used for the treatment of allergic rhinitis?	×			×		×
Should oral H1 antihistamines vs. no treatment be used for the treatment of allergic rhinitis?	×			×		×
Should intraocular H1 antihistamines vs. no treatment be used for the treatment of ocular symptoms in patients with allergic rhinitis?	×			×		×
Should intranasal glucocorticosteroids vs. no treatment be used for the treatment of allergic rhinitis?	×			×		×
Should oral leukotriene‐receptor antagonists vs. no treatment be used for the treatment of allergic rhinitis?	×			×		×
Should intranasal decongestants vs. no treatment be used for the treatment of allergic rhinitis?	×			×		×
Should new‐generation oral H1 antihistamines vs. old‐generation oral H1 antihistamines be used for the treatment of allergic rhinitis?	×					×
Should intranasal H1 antihistamines vs. intranasal glucocorticosteroids be used for the treatment of allergic rhinitis?	×			×		×
Should intranasal H1 antihistamines vs. oral H1 antihistamines be used for the treatment of allergic rhinitis?	×					×
Should intranasal H1 antihistamines vs. intranasal chromones be used for the treatment of allergic rhinitis?	×					×
Should intranasal glucocorticosteroids vs. oral H1 antihistamines be used for the treatment of allergic rhinitis?	×				×	×
Should a combination of an intranasal H1 antihistamine and an intranasal glucocorticosteroid vs. an intranasal glucocorticosteroid alone be used for the treatment of allergic rhinitis?	×					×
Should a combination of an intranasal H1 antihistamine and an intranasal glucocorticosteroid vs. an intranasal H1 antihistamine alone be used for the treatment of allergic rhinitis?	×					×
Should a combination of an oral H1 antihistamine and an intranasal glucocorticosteroid vs. an intranasal glucocorticosteroid alone be used for the treatment of allergic rhinitis?	×				×	×
Should a combination of an oral H1 antihistamine and an intranasal glucocorticosteroid vs. an oral H1 antihistamine alone be used for the treatment of allergic rhinitis?		×				×
Should a combination of an oral leukotriene‐receptor antagonist and an oral H1 antihistamine vs. an oral H1 antihistamine alone be used for the treatment of allergic rhinitis?		×				×
Should any specific individual intranasal glucocorticosteroid vs. other individual intranasal glucocorticosteroids be used for the treatment of allergic rhinitis?		×				×
Should a monoclonal antibody vs. no treatment be used for the treatment of patients with moderate to severe allergic rhinitis?		×				×
Should masks vs. no masks be used for preventing exposure to air pollution and other triggers in patients with allergic rhinitis?		×				×
Should air purifiers vs. no air purifiers be used for the management of patients with allergic rhinitis?		×			×	×
Should patient diaries vs. no patient diaries be used for monitoring in patients with allergic rhinitis?		×				×
Should digital patient diaries vs. nondigital patient diaries be used for monitoring elderly patients with allergic rhinitis?			×			×
Should a dual approach combining both long‐term and short‐term monitoring vs. long‐term monitoring alone be used in patients with allergic rhinitis?			×			×
Should a systematic diagnostic screening for asthma vs. no systematic screening be used in patients with allergic rhinitis?			×		×	×
Should a systematic diagnostic screening for allergic rhinitis vs. no systematic screening be used in patients with asthma?			×			×
Should a systematic assessment of the effect of allergic rhinitis on quality of life, work productivity, and/or school performance vs. no systematic assessment be used in patients with allergic rhinitis?			×			×
Should therapy adherence monitoring vs. no therapy adherence monitoring be used in treated patients with allergic rhinitis?		×				×
Should modern methods to increase adherence (e.g., shared decision‐making, guided self‐management) vs. traditional methods to increase adherence be used for the management of patients with allergic rhinitis?		×				×
Should patient education and self‐management strategies vs. no patient education and self‐management strategies be used in patients with allergic rhinitis?		×			×	×
Should any specific individual oral H1 antihistamine vs. other individual oral H1 antihistamines be used for the treatment of allergic rhinitis?				×		×
Should a combination of an intranasal H1 antihistamine and an intranasal glucocorticosteroid vs. no treatment be used for the treatment of allergic rhinitis?				×		×
Should intraocular H1 antihistamines vs. oral H1 antihistamines be used for the treatment of ocular symptoms in patients with allergic rhinitis?					×	×
Should a systematic diagnostic screening for nonallergic triggers (such as irritants or pollutants) vs. no systematic screening be used in patients with allergic rhinitis?					×	×
Should a systematic diagnostic screening for sinusitis vs. no systematic screening be used in patients with allergic rhinitis?					×	×
Should digital platforms (like smart watches, fitness trackers and mobile apps) vs. no digital platforms be used in patients with allergic rhinitis?					×	×
Should oral glucocorticosteroids vs. no treatment be used for the treatment of allergic rhinitis?	×			×		
Should intramuscular glucocorticosteroids vs. no treatment be used for the treatment of allergic rhinitis?	×					
Should intranasal chromones vs. no treatment be used for the treatment of allergic rhinitis?	×					
Should intraocular chromones vs. no treatment be used for the treatment of ocular symptoms in patients with allergic rhinitis?	×					
Should intranasal ipratropium bromide vs. no treatment be used for the treatment of allergic rhinitis?	×			×	×	
Should oral decongestants vs. no treatment be used for the treatment of allergic rhinitis?	×			×		
Should intranasal glucocorticosteroids vs. oral leukotriene‐receptor antagonists be used for the treatment of allergic rhinitis?	×				×	
Should oral leukotriene‐receptor antagonists vs. oral H1 antihistamines be used for the treatment of allergic rhinitis?	×				×	
Should oral leukotriene‐receptor antagonists vs. intranasal H1 antihistamines be used in patients with allergic rhinitis?		×			×	
Should intraocular H1 antihistamines vs. nasal H1 antihistamines be used for the treatment of ocular symptoms in patients with allergic rhinitis?		×				
Should a combination of an intranasal decongestant and an oral H1 antihistamine vs. an oral H1 antihistamine alone be used for the treatment of allergic rhinitis?		×				
Should a combination of oral decongestants and H1 antihistamines vs. oral H1 antihistamines alone be used for the treatment of allergic rhinitis?	×			×		
Should nasal irrigation alone vs. no treatment be used for the treatment of allergic rhinitis?			×	×	×	
Should nasal irrigation as an add‐on to pharmacologic treatment vs. pharmacologic treatment alone be used for the treatment of allergic rhinitis?			×			
Should nasal barriers (e.g., petroleum jelly) vs. no nasal barriers be used for preventing exposure to air pollution in patients with allergic rhinitis?		×				
Should sunglasses vs. no sunglasses be used for preventing exposure to air pollution and other triggers in patients with allergic rhinitis?		×				
Should temperature‐controlled laminar airflow devices vs. no temperature‐controlled laminar airflow devices be used for the management of patients with allergic rhinitis?		×				
Should drying bed linen and clothes indoors vs. drying bed linen and clothes outdoors be used for preventing exposure to pollen in patients with allergic rhinitis?		×			×	
Should anti‐Fel d1 cat food vs. regular cat food be used in cats of patients with allergic rhinitis with allergies to cats?			×			
Should early warning systems vs. no early warning systems be used in patients with allergic rhinitis?		×				
Should a routine olfactory test vs. no routine olfactory test be used for the monitoring of patients with allergic rhinitis?		×				
Should a systematic assessment of disease control vs. a systematic assessment of disease severity be used in patients with allergic rhinitis?		×				
Should a systematic diagnostic screening for conjunctivitis vs. no systematic screening be used in patients with allergic rhinitis?		×				
Should a systematic screening for local allergic rhinitis vs. no systematic screening for local allergic rhinitis be used in patients with rhinitis clinically triggered by airborne allergens but with negative results on skin prick tests and specific IgE to environmental allergens?		×				
Should pharmacologic treatment in the morning vs. pharmacologic treatment in the evening be used for the treatment of allergic rhinitis?				×		
Should intraocular decongestants vs. no treatment be used for the treatment of ocular symptoms in patients with allergic rhinitis?				×		
Should more than 1 daily oral H1 antihistamine vs. 1 single daily oral H1 antihistamine be used for the treatment of allergic rhinitis?				×		
Should H1 antihistamines vs. decongestants be used for the treatment of allergic rhinitis?				×		
Should any specific individual intranasal decongestant vs. other individual intranasal decongestants be used for the treatment of allergic rhinitis?				×	×	
Should any specific individual oral decongestant vs. other individual oral decongestants be used for the treatment of allergic rhinitis?				×		
Should any specific individual intranasal H1 antihistamine vs. other individual intranasal H1 antihistamines be used for the treatment of allergic rhinitis?				×		
Should any specific individual intraocular H1 antihistamine vs. other individual intraocular H1 antihistamines be used for the treatment of ocular symptoms in patients with allergic rhinitis?				×		
Should a combination of oral decongestants and acetaminophen vs. oral decongestants alone be used for the treatment of allergic rhinitis?				×		
Should a combination of oral H1 antihistamines and acetaminophen vs. oral H1 antihistamines alone be used for the treatment of allergic rhinitis?				×		
Should a combination of oral H1 antihistamines and expectorant medications (mucoactive agents) vs. oral H1 antihistamines alone be used for the treatment of allergic rhinitis?				×		
Should inhaled glucocorticosteroids (antiasthmatics) vs. no treatment be used for the treatment of allergic rhinitis?				×		
Should cough suppressants vs. no treatment be used for the treatment of allergic rhinitis?				×		
Should expectorant medications (mucoactive agents) vs. no treatment be used for the treatment of allergic rhinitis?				×		
Should acetaminophen vs. no treatment be used for the treatment of allergic rhinitis?				×		
Should nonsteroidal anti‐inflammatory drugs vs. no treatment be used for the treatment of allergic rhinitis?				×		
Should decongestant topical ointments vs. no treatment be used for the treatment of allergic rhinitis?				×		
Should specific sleep positions vs. other sleep positions be used for the management of allergic rhinitis?				×		
Should humidifiers vs. no intervention be used for the management of allergic rhinitis?				×		
Should nasal strips vs. no intervention be used for the management of allergic rhinitis?				×		
Should intranasal decongestants vs. oral decongestants be used for the treatment of allergic rhinitis?					×	
Should intranasal ipratropium bromide vs. intranasal glucocorticosteroids be used for the treatment of allergic rhinitis?					×	
Should psychological support or stress management techniques vs. no psychological intervention be used in patients with allergic rhinitis?					×	
Should tailored advice on physical activity vs. no specific advice be used in patients with allergic rhinitis?					×	
Should a systematic diagnostic screening for sleep disturbances, including sleep apnea, vs. no systematic screening be used in patients with allergic rhinitis?					×	
Should telemedicine vs. in‐person visits alone be used in patients with allergic rhinitis?					×	

Abbreviations: ARIA, Allergic Rhinitis and its Impact on Asthma; KOL, key opinion leaders; LLM, large language models.

### 4.1 Development of Targeted Guideline Questions

#### 4.1.1 Building on Established Knowledge: Questions From Previous ARIA Guidelines (Physician‐Centred Approach)

We retrieved all questions regarding the pharmacological and non‐pharmacological treatment of AR that had been answered in ARIA 2010 and 2016 and the US Practice Parameters [39].

#### 4.1.2 Insights From Experts: Questions From Panel Members (Key Opinion Leader‐Centred Approach)

We surveyed guideline panel members on relevant questions regarding the pharmacological or non‐pharmacological treatment of AR. To avoid duplicate questions, we provided panel members with the list of guideline questions that had been answered in the previous ARIA guidelines or identified through analysis of MASK‐air studies.

#### 4.1.3 Leveraging Direct Patient‐Reported Data: Questions From MASK‐air Studies (Person‐Centred Approach)

To formulate questions based on observations of day‐to‐day practice and patients' experience, two guideline methodologists (BSP and RJV) (i) read all the original studies that used MASK‐air data and (ii) formulated guideline questions based on the main messages and hypotheses raised by these studies.

#### 4.1.4 AI‐Supported Development of Guideline Questions (Person‐Centred Approach)

To better reflect patients' beliefs and needs, AI was used in the development of guideline questions [16]. We retrieved popular queries on AR (identified using Google Trends) and used ChatGPT 4.0 to classify them into those conveying potentially relevant questions or not [16]. Queries identified as potentially conveying relevant questions were then manually transformed into guideline questions.

We also prompted ChatGPT 4.0 to assume the role of a patient or of a healthcare provider and to generate potentially relevant guideline questions [16].

### 4.2 Prioritisation of Guideline Questions

Developed guideline questions were voted for prioritisation by the guideline panel members who rated the priority of each question on a scale of 1–9 (9 indicating highest priority) [40] according to the criteria and signalling questions displayed in Box S1. For a question to be prioritised, a mean priority rate of at least 6.3 or a median rate of at least 7 was required.

Among the 89 unique developed questions, 39 were voted by the guideline panel members as “prioritised questions”. Non‐prioritised questions were sent to the ARIA review group. The review group could recommend prioritising some of those questions or even propose new questions of interest. Overall, three additional questions were added further to feedback from the review group.

## 5 Identification and Prioritisation of the Outcomes

For the formulation of guideline recommendations, interventions need to be judged in terms of their desirable and undesirable effects. This implies assessing the effect of interventions on a set of pre‐selected outcomes (ideally not more than seven).

In ARIA‐EAACI 2024–2025, a set of potentially relevant outcomes was identified by considering (i) an initial proposal by the guideline co‐chairs, (ii) outputs from a large language model, (iii) subsequent suggestions by guideline panel members and (iv) a systematic review of patients' values. For each identified outcome, we developed health outcome descriptors to create common definitions that describe the outcomes with respect to symptoms, time horizon, testing and treatment and consequences [41] (example in Box S2). This ensured that panel members displayed a common understanding when discussing each of the outcomes.

Guideline panel members were then asked to rate the priority of each outcome on a scale of 1–9 (9 indicating the highest priority). Of the 28 outcomes voted for prioritisation, the five rated as being of highest priority were nasal symptoms, quality‐of‐life impairment, eye symptoms, total symptoms and serious adverse events (AEs). Since these top outcomes only included one undesirable effect (serious AEs), we considered a sixth outcome corresponding to the occurrence of any AE. The latter corresponded to the undesirable effect outcome with the second highest priority rating.

## 6 Emphasising Diverse Populations

### 6.1 Age Groups

For each guideline recommendation, we provide subgroup considerations for children. To gather evidence on the effectiveness and safety of AR treatments in children, we conducted systematic reviews solely assessing the paediatric population.

Between adolescents and adults, similar AR control levels were found using evidence from MASK‐air [42]. On the other hand, there seem to be differences for individuals over 75 years of age [43], despite the lack of available evidence.

### 6.2 Sex

Sex impacts health, with relevant effect modification and implications on the prevention, screening, diagnosis and treatment of allergic diseases [44]. This is particularly true during puberty and pregnancy [45, 46, 47]. However, since most studies do not provide evidence on the effect of interventions according to sex, more data are needed to incorporate sex‐specific considerations in the guidelines.

### 6.3 Patients With Multimorbid Asthma and/or Conjunctivitis

There is mounting evidence that AR alone and AR + asthma multimorbidity represent two distinct phenotypes [48] (Table S3). Such evidence—obtained from the MeDALL (Mechanisms of the Development of Allergy) study [49] and MASK‐air data [50]—is grounded on insights into polysensitisation and multimorbidity, advances in mHealth for novel phenotype definition, confirmation in canonical epidemiologic studies and genomic findings. Therefore, subgroup considerations for patients with asthma were provided in the guidelines whenever justified.

Differences between AR alone and AR + conjunctivitis were identified with MASK‐air data [51, 52, 53, 54] and confirmed in canonical epidemiologic studies. These studies have shown that ocular symptoms (i) are more common in AR + asthma than in AR alone [54], (ii) are associated with the severity of nasal symptoms [55, 56] and (iii) are important to consider in severe asthma [55]. These data indicate that conjunctivitis should be considered as a separate disease in AR or AR + asthma and should be embedded in the multimorbid phenotype. However, more data are needed to incorporate this phenotype in guidelines.

## 7 Obtaining Evidence for Evidence‐to‐Decision Frameworks

Recommendations were generated using EtDs [11, 12]. In its standard form, the EtD comprises 12 criteria, including problem priority, desirable effects, undesirable effects, certainty of evidence [in desirable and undesirable effects], values and preferences, balance of effects, resources required, certainty of evidence of required resources, cost‐effectiveness, equity, acceptability and feasibility [11, 12]. In addition, in ARIA‐EAACI 2024–2025, a 13th criterion, Planetary Health, was considered [57]. The next subsections of this paper will discuss the evidence sources used to inform each EtD criterion. An example of an EtD (comparison between fixed combinations of INCS + intranasal antihistamines [INAH] vs. INCS) is available in the Appendix S1.

### 7.1 Desirable and Undesirable Effects of Interventions

#### 7.1.1 Pairwise and Network Meta‐Analyses of Randomised Controlled Trials

There was insufficient evidence regarding the comparative efficacy and safety of pharmacological treatments of AR before the beginning of this project. We therefore decided to conduct systematic reviews with pairwise or network meta‐analyses to inform the comparative effectiveness of treatments [58]. Overall, for informing ARIA 2024–2025, we planned to conduct six systematic reviews of RCTs comparing the desirable and undesirable effects of oral and intranasal medications:

- Systematic review and pairwise meta‐analysis comparing intranasal medications versus placebo in adults;
- Systematic review and network meta‐analysis comparing intranasal medications in adults;
- Systematic review and network meta‐analysis comparing intranasal medications in children;
- Systematic review and pairwise meta‐analysis comparing intranasal versus oral medications in adults;
- Systematic review and network meta‐analysis comparing individual oral medications in adults and children;
- Systematic review and network meta‐analysis comparing classes of intranasal and oral medications in adults and children.

Three systematic reviews [59, 60, 61] and two protocols [62, 63] have been published. In line with the prioritised outcomes for ARIA‐EAACI 2024–2025, these systematic reviews assessed the effects of each individual medication or medication class on nasal symptoms, ocular symptoms, rhinoconjunctivitis‐related quality of life and AEs. Pairwise and network meta‐analyses including both active comparisons and comparing medications with placebo provided information on untreated patients [59, 60, 61]. Network meta‐analyses of active comparisons provided information on treated patients [59].

As for the main results of the published studies, both the pairwise and the network meta‐analysis on intranasal medications have found that azelastine‐fluticasone, fluticasone furoate and fluticasone propionate were the medications with the highest probability of resulting in moderate or large improvements in the assessed outcomes [59, 60]. Considering the differential impact on ocular symptoms, fluticasone furoate or fluticasone propionate may be particularly indicated for patients with severe nasal symptoms but no or mild ocular symptoms, while azelastine‐fluticasone may be an adequate option for patients with moderate to severe ocular symptoms.

In addition, the network meta‐analysis on intranasal medications found that azelastine‐fluticasone and fluticasone furoate were the medications with the highest probability of being the most efficacious, as they frequently resulted in clinically meaningful larger improvements when compared to other active treatments [59]. In children, evidence was scarcer than in adults. However, despite being efficacious and safe, the efficacy of AR treatments in children does not seem to be as high as in adults.

On the other hand, the meta‐analysis comparing intranasal versus oral medications provided evidence that intranasal medications are more effective than oral ones (namely, oral antihistamines or oral leukotriene‐receptor antagonists) [61].

Differences in the frequency of undesirable effects (AEs or serious AEs) were trivial for most comparisons between individual medications or between different medication classes [59, 61].

#### 7.1.2 Pharmacovigilance Data

Considering limitations of RCTs in registering AEs (e.g., limited number of participants, short period of follow‐up and restricted eligibility criteria) [64, 65], we analysed pharmacovigilance data to provide further information on the undesirable effects of interventions. We queried the VigiBase database on reported AEs to individual medications of different classes used for the treatment of AR. VigiBase, the World Health Organization global database of AE reports for medicines and vaccines, is the largest pharmacovigilance database [66]. The analysis of its data allowed us to identify the 15 most commonly reported AEs for each intervention being compared in each question. For each AE, we calculated the respective reporting odds ratio to identify signals of disproportionate reporting [67]. For example, in the comparison between INCS and INAH, we found that cataract and glaucoma were disproportionally more commonly reported with INCS than with INAH.

### 7.2 Values

Healthcare interventions typically result in benefits and harms. Values concern the relative importance patients place on specific benefits and harms [38, 68]. We performed a systematic review to provide a comprehensive overview of the values of patients with AR [38]. We observed that (i) patients generally value the efficacy of interventions more than their AEs, and (ii) patients consider nasal symptoms (particularly nasal obstruction) as those with the highest impact. One of the included studies of that systematic review was based on direct patient data from MASK‐air, involving the computation of utilities in different European countries according to the level of rhinitis control and the presence of comorbid asthma [69]. The results of the study were coherent with a questionnaire sent to ARIA members [70].

### 7.3 Resources Required and Cost‐Effectiveness

#### 7.3.1 Global Survey on Medication Costs

**FIGURE 3:**
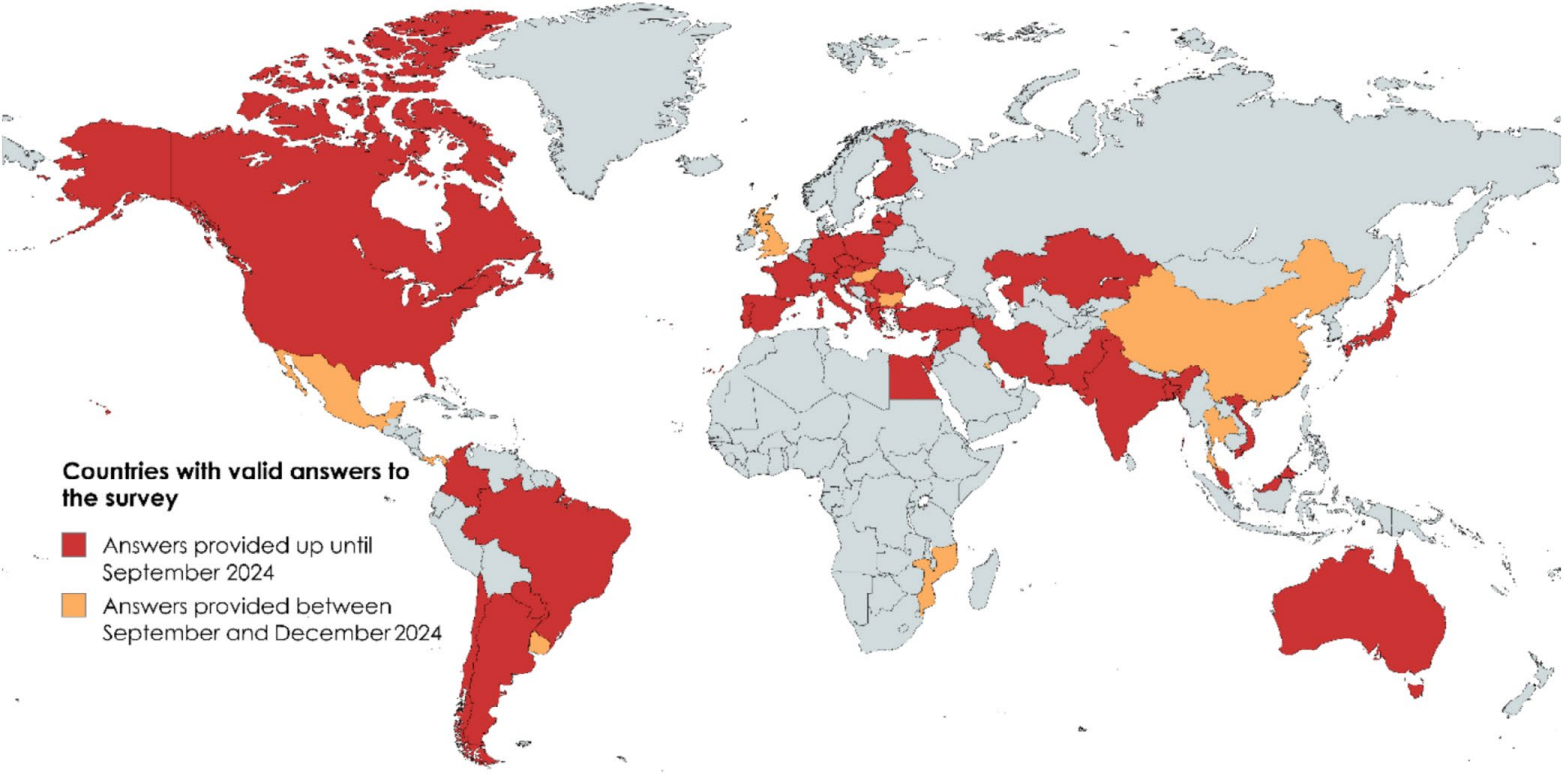
Countries with valid answers to the survey sent to ARIA experts on the availability and costs of allergic rhinitis medication. The answers provided up until September 2024 were used to inform the ARIA‐EAACI 2024–2025 guidelines.

The cost of medications is highly variable across countries. Moreover, medication costs change with time, particularly when generic drugs are made available or when medications become over‐the‐counter. Therefore, to gather information on this criterion, we conducted a survey asking ARIA experts about the availability and lowest costs of individual rhinitis medications in their countries (Figure 3).

#### 7.3.2 MASK‐air Data on Indirect Costs Associated With Rhinitis Control

We used data from MASK‐air to quantify indirect costs resulting from productivity losses associated with worse rhinitis control. MASK‐air includes WPAI:AS, a validated questionnaire that measures the impact of allergies on absenteeism and presenteeism [32]. Based on these data, we estimated the underlying indirect costs in 40 countries according to the level of rhinitis control [71]. Overall, median work impairment (including both absenteeism and presenteeism) stood at 4.6% (P25‐P75 = 0.8%–15.1%) for well‐controlled weeks, ascending to 27.7% (P25‐P75 = 12.4%–46.0%) and 60.7% (P25‐P75 = 38.5%–80.2%) for partially and poorly controlled weeks, respectively. For example, in Germany, this translates into median weekly monetary losses of 42.3 US dollars (US$) adjusted for purchasing power parity (PPPs) for well‐controlled weeks, 259.7 US$ PPPs for partially controlled weeks, and 554.2 US$ PPPs for poorly controlled weeks (difference of 511.9 US$ per week from poor to good control). For other Western European countries, the difference ranges from 200 to 695 US$ [71].

#### 7.3.3 “Cost‐Utility” Assessments Based on Health Technology Assessment Reports and MASK‐air Data

There is a scarcity of recent cost‐effectiveness and cost‐utility studies on AR treatments. We therefore considered cost data from the survey to ARIA experts and either (i) utilities data estimated from a health technology assessment report [72] or (ii) MASK‐air data on levels of the VAS of EQ‐5D associated with each intervention. The use of VAS EQ‐5D data reported in MASK‐air was justified since (i) the identified health technology assessment report did not assess all medication classes and (ii) there is a lack of available studies estimating utilities associated with different treatments for AR.

### 7.4 Equity

#### 7.4.1 Global Survey on the Availability of Medications

To assess across‐country differences in the availability of medications for AR, we conducted a survey asking ARIA experts about the availability of individual rhinitis medications in their countries (Figure 3).

#### 7.4.2 World Health Organization List of Essential Medicines

We assessed the medications for AR included in the World Health Organization List of Essential Medicines [73]. Only intranasal budesonide, oral loratadine and intranasal xylometazoline are on this list. According to information from local experts, medications available in this list are provided free of charge in some low‐income countries.

### 7.5 Acceptability and Feasibility

#### 7.5.1 Assessment of Patient Satisfaction With Treatment in MASK‐air Data

Patients' satisfaction with their treatment is a patient‐reported outcome measure distinct from quality of life and symptoms [74]. In its daily monitoring questionnaire, the MASK‐air app includes a question assessing (through a 0–100 VAS) how satisfied patients are with their rhinitis treatment on that day. We have analysed VAS satisfaction data for each treatment class or individual medication in single medication and in co‐medication. In addition, we have built multivariable regression models to compare different medications on VAS satisfaction levels.

#### 7.5.2 Assessment of Co‐Medication Use in the MASK‐air Data

We assessed MASK‐air data to compare different individual medications and medication classes on the frequency of their use in co‐medication. MASK‐air studies have consistently found that when patients use co‐medication, they tend to report more severe rhinitis symptoms [18, 75]. This finding may be explained by the fact that patients tend to increase their medication use when feeling less well controlled.

#### 7.5.3 Assessment of Patients' Adherence to Treatment in the MASK‐air Data

RCTs tend to assess strategies involving the daily use of medications and, henceforth, require a high adherence to treatment (usually 70%). However, in MASK‐air, two studies have addressed adherence to rhinitis treatment and have found that most AR patients are not adherent [76, 77]. In particular, most patients use treatment on an as‐needed basis when feeling symptomatic [18]. Therefore, to inform about the acceptability of interventions, we analysed MASK‐air data to compare the adherence to different rhinitis medication classes.

#### 7.5.4 Speed of Onset of Action of Medications: Rapid Evidence Review

We conducted a rapid evidence review to assess the speed of onset of action of AR medications based on ARIA 2019 and other sources [9]. We took into account the guideline published by the European Medicines Agency on the “clinical development of medicinal products for the treatment of allergic rhinoconjunctivitis” [78], and considered the onset of action of medications. Evidence on the onset of action of medications for AR may originate from three study types: standard phase III double‐blind RCTs, park setting studies (natural exposure) and allergen exposure chamber studies (artificial exposure). In this rapid review, we found that medications containing INAH act within 5–30mins, whereas most INCS need hours to be effective.

### 7.6 Planetary Health

The concept of Planetary Health emphasises the inherent connection between the health of humans and the health of the planet, on which human health depends [79]. There is increasing understanding that clinical decision guidelines need to incorporate a planetary health dimension [80]. The consideration of Planetary Health in ARIA started with the participation in an EU High‐Level meeting (Finnish Presidency of the European Union and DG Research) on Planetary Health [81, 82, 83].

ARIA‐EAACI 2024–2025 considered aspects of Planetary Health in the formulation of guideline recommendations by having Planetary Health criteria in the EtD [57, 80]. For example, for comparisons between intranasal versus oral treatments, aspects such as the global warming potential of the different packaging types and manufacturing places were considered for available evidence.

## 8 Issuing Judgements and Actionable, Patient‐Centred Recommendations for Each Guideline Question

In the EtDs, evidence is provided for each criterion, so that the guideline panel can make a judgement. Based on the judgements provided for all criteria, the panel is then able to issue a recommendation for the respective guideline question.

The ARIA‐EAACI 2024–2025 guideline panel held regular online meetings to make judgements on the EtD criteria and formulate guideline recommendations. In each case, consensus among members of the panel without CoI was sought. If consensus was not reached, a formal voting process was set (using the anonymous voting feature of GRADEpro), although restricted to guideline panel members without CoI.

We drafted recommendations as suggested by the GRADE working group. GRADE recommendations are characterised by (i) a directionality (i.e., whether there is favouring of the intervention, of the comparison, or of neither the intervention nor the comparison) and (ii) a strength (i.e., whether the recommendation is strong or conditional). Strong recommendations posit that the intervention should be applied to most individuals. Conditional recommendations indicate that the recommendation may not be applicable to relevant subsets of patients and that variability in the clinical practice may be apropriate [84, 85]. In addition, recommendations should inform on the underlying certainty of evidence (quality of the whole body of evidence, considering altogether desirable and undesirable effects).

After drafting guideline recommendations, the guideline panel proposes subgroup considerations for children and, if justified, patients with asthma. If necessary, the guideline panel also (i) suggests implementation considerations (e.g., in aspects related to the application of recommendations in low‐ and middle‐income countries), (ii) discusses aspects related to monitoring and evaluation and/or (iii) highlights related research priorities.

## 9 From Recommendations to Action: Building Digitalised Decision Algorithms

In ARIA 2010–2016, a simple algorithm was proposed in treated and untreated patients. In ARIA‐EAACI 2024–2025, we will propose more management algorithms, considering a diverse set of scenarios in terms of patients (e.g., controlled and treated, uncontrolled and treated or uncontrolled and untreated), settings (e.g., primary care or specialised allergy care) or regions (e.g., low‐ and middle‐income countries or high‐income countries; the survey we conducted among ARIA experts will support such tailoring, as it informs on the availability and price of medication in different countries). The scenarios will be jointly proposed by the guideline panel group, the review group and ARIA Junior members. The corresponding algorithms will be built based on the formulated recommendations.

**FIGURE 4:**
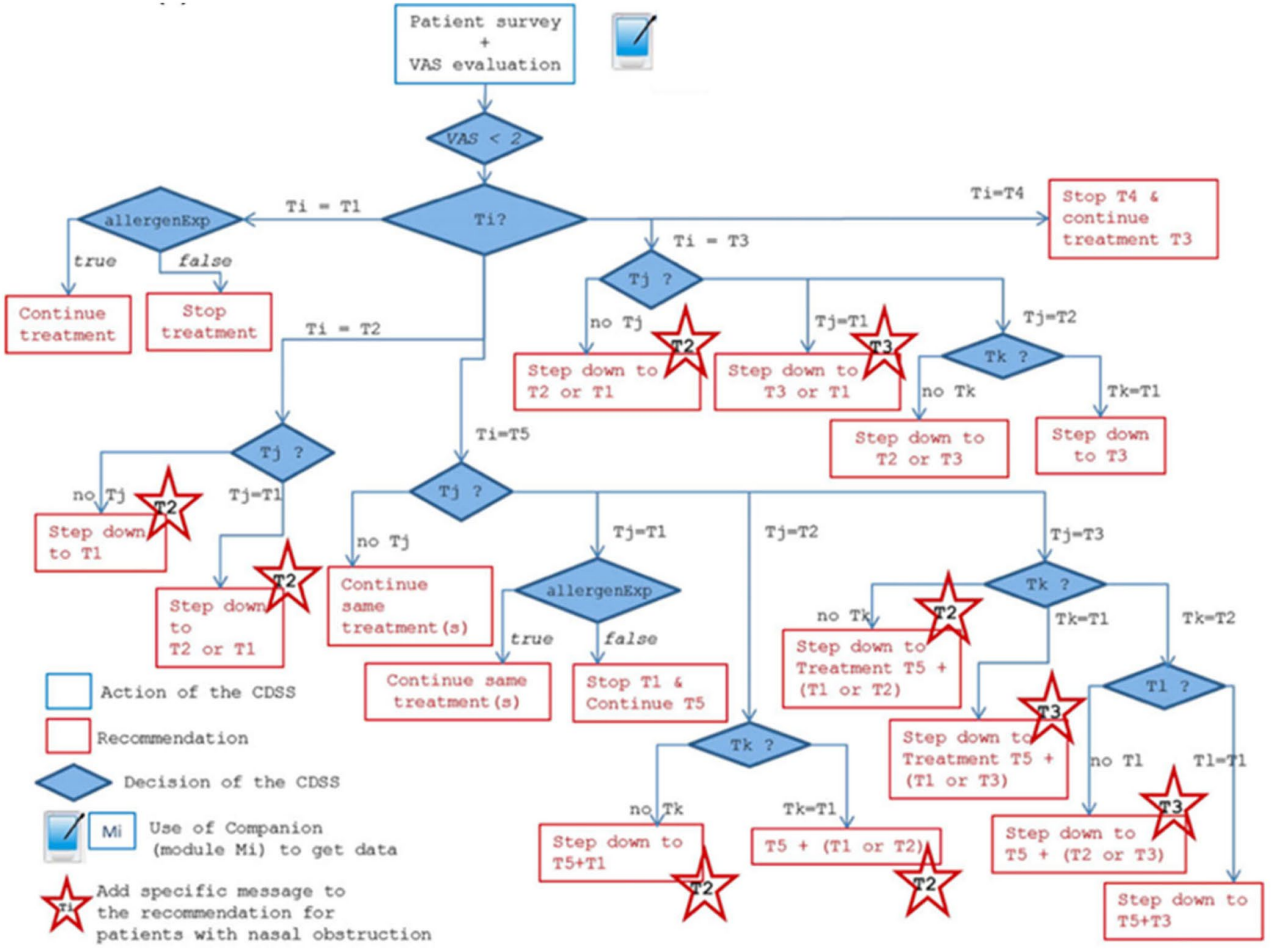
Example of an electronic clinical decision support system (ARIA 2016) (example for patients with well‐controlled allergic rhinitis). Ti = Class of current treatment (in case of polypharmacy, Ti = maximum class); Tj, Tk and Tl = Medications added to Ti, order of class l < k < j < i. T1 = antihistamine (oral, intranasal and eye drops), leukotriene‐receptor antagonist, chromone (intranasal and eye drops); T2 = intranasal corticosteroid (INCS); T3 = INCS+azelastine; T4 = add short course of oral corticosteroids; T5 = consider referral and allergen‐specific immunotherapy.

In ARIA 2010–2016, an algorithm was developed [14]. Moreover, an electronic clinical decision system provided evidence‐based treatment recommendations. The app interfaces used to collect the necessary information had been described, and the management algorithms had been programmed into the MASK‐air app using Titanium Appcelerator for iOS tablets [86] (Figure 4 and Figure S1). However, since MASK‐air was not at that time an MDR Class IIa, the digitalised algorithms were not implemented. In ARIA 2024–2025, the developed management algorithms will be digitalised and implemented in MASK‐air.

## 10 Conclusions

ARIA‐EAACI 2024–2025 will provide major advances for the management of patients with AR. Building on several different sources of evidence, ARIA‐EAACI 2024–2025 will adopt a patient‐centred perspective and promote shared decision‐making. The formulation of recommendations will be fully based on the GRADE working group methodology. Nevertheless, the development of ARIA‐EAACI 2024–2025 will contribute per se to methodological innovations for the guideline development process in aspects such as the use of AI to support the development of questions, or the incorporation of evidence from multiple complementary sources (Table 1). As its end product, ARIA‐EAACI 2024–2025 aims not only at producing a set of recommendations but also at developing a set of digitalised management algorithms, which will be essential in promoting a continuative and long‐standing integration of these guidelines into daily care.

## Conflicts of Interest Management

Conflicts of interest (CoIs) of all participants were managed according to the Guidelines International Network (GIN) CoI declaration and management principles reviewed by the AWMF (*Arbeitsgemeinschaft der Wissenschaftlichen Medizinischen Fachgesellschaften e. V*.) [87]. A CoI was defined as an individual having “current material interests outside of ARIA that could influence or could be perceived as influencing his/her decisions, actions, or presentations.”

Before participation, all guideline panel members and members of the evidence‐synthesis team completed a standardised disclosure‐of‐interests (DoI) form provided by the AWMF. This form collected information about current and previous (past 3 years) interests in the healthcare sector, including direct financial interests and indirect (e.g., non‐financial and intellectual) interests. Guideline panel members were required to report all interests in the DoI form, regardless of whether they would see such interests as having a thematic reference to the guideline or representing a conflict of interest.

Staff providing administrative support to ARIA‐EAACI 2024–2025 judged every DoI form in the assessment on whether a disclosed interest was considered a CoI. The completed DoI forms are to be made available to all panel members during the project. At every meeting, guideline panellists were reminded of the DoI forms and prompted to make new disclosures. ARIA staff and members reviewed new disclosures, but previously documented disclosures or judgments are never removed or changed. The forms will receive a final update prior to guideline submission and will be included with the published guidelines.

The guideline panel was composed so that most of its members did not have a CoI. Absence of CoI is required for those chairing the panel discussions and gathering and summarising evidence for the EtDs. No individual was excluded from the guideline panel due to direct or indirect CoI. However, only those without CoI were allowed to make judgements or participate in the voting process for judgements or recommendations.

## Conflicts of Interest

J. Bousquet reports personal fees from Cipla, Menarini, Mylan, Novartis, Purina, Sanofi‐Aventis, Teva, Noucor, other from KYomed‐Innov, other from Mask‐air‐SAS, outside the submitted work. M. Hyland reports grants from GSK, from AstraZeneca, from null, outside the submitted work. A. Cruz reports grants, personal fees and non‐financial support from AstraZeneca, personal fees from Chiesi, grants and personal fees from Eurofarma, personal fees from Farmoquimica, grants from EMS, grants and personal fees from GSK, grants and personal fees from Sanofi, outside the submitted work. J. Sastre reports grants and personal fees from Sanofi, personal fees from GSK, personal fees from Novartis, personal fees from AstraZeneca, personal fees from Mundipharma and personal fees from Faes Farma, outside the submitted work. D. Wallace reports as an author on the JTFPP practice parameters on Rhinitis, the most recent being 2020 and I have been an author on several previous ARIA and MASK‐related articles. M. Wagenmann reports personal fees from Allergopharma, personal fees from ALK‐Abello, grants and personal fees from AstraZeneca, personal fees from CSL Behring, grants and personal fees from GSK, personal fees from HAL Allergy, personal fees from Leti Pharma, personal fees from M.S.D., personal fees from Novartis, grants and personal fees from Regeneron, grants and personal fees from Sanofi, personal fees from Stallergenes, grants from Takeda, outside the submitted work. O. Pfaar reports grants and/or personal fees and/or travel support from AEDA, Alfried Krupp Krankenhaus, ALK‐Abelló, Allergopharma, Almirall, Altamira Therapeutics, ASIT Biotech, AstraZeneca, Bencard Allergie GmbH/Allergy Therapeutics, Blueprint, Cliantha, Deutsche AllergieLiga e.V., Deutsche Forschungsgemeinschaft, Dustri‐Verlag, ECM Expro & Conference Management GmBH, Forum für Medizinische Fortbildung, Georg‐Thieme‐Verlag, GSK, HAL Allergy Holding B.V./HAL Allergie GmbH, Immunotek, Ingress Health, Institut für Disease Management (Essen, Germany), IQVIA Commercial, Japanese Society of Allergology, Königlich Dänisches Generalkonsulat, Laboratorios LETI/LETI Pharma, Lilly, Lofarma, Medizinische Hochschule Hannover, med update europe GmbH, Meinhardt Congress GmbH, Novartis, Paul‐Ehrlich‐Institut, Paul‐Martini‐Stiftung, PneumoLive, Pohl‐Boskamp, Procter & Gamble, Red Maple Trials Inc., R.G. Aerztefortbildung, ROXALL Medizin, Sanofi‐Aventis, Sanofi Genzyme, Springer Publisher, Stallergenes Greer, streamedup! GmbH, Technical University Dresden, Wiley Publishers, Wort & Bild Verlag, Verlag ME; outside the submitted work, Oliver Pfaar is Vice President of the European Academy of Allergy and Clinical Immunology (EAACI), a member of EAACI Excom as well as a member of the external board of directors of the German Society of Allergy and Clinical Immunology (DGAKI); coordinator, main‐ or co‐author of different position papers and guidelines in rhinology, allergology and allergen immunotherapy; and he is Editor‐in‐Chief of Clinical Translational Allergy and Associate Editor of Allergy. T. Iinuma reports a grant from Sanofi, outside the submitted work. J. Davies reports grants from Australian National Health and Medical Research Council, grants from Australian Research Council, non‐financial support from Sullivan Nicolaides Pathology Queensland, non‐financial support from ThermoFisher Sweden, non‐financial support from Swisens SA Switzerland, non‐financial support from Kenelec Australia, non‐financial support from Abacus Dx Australia, grants from Bayer Healthcare, grants from Australian Department of Health and Ageing, grants from Australian Medical Research Future Fund, outside the submitted work; in addition, Dr. Davies has a patent US PTO 14/311944 issued, a patent AU2008/316301 issued and a patent PCT/AU2024/051221 pending. I. Tsiligianni reports grants from Chiesi, GSK Hellas, Menarini, AstraZeneca Greece, outside the submitted work. B. Gradauskiene reports grants from AstraZeneca, personal fees from Berlin‐Chemie Menarini, personal fees from Viatris, outside the submitted work. I. Ansotegui reports personal fees from Bayer, personal fees from Eurodrug, personal fees from Gebro, personal fees from Menarini, personal fees from M.S.D., personal fees from Roxall, personal fees from Sanofi, personal fees from Cipla, personal fees from Glenmark, personal fees from Opella, outside the submitted work. A. Fiocchi reports Danone Advisory board member, Abbott—Consultant, paid to institution, Aimmune—Advisory board member, Ferrero—Consultant, Novartis—Advisory board member, Sanofi—Advisory board member, Stallergenes—Advisory board member. M. Kupczyk reports personal fees from Adamed, personal fees from Abbvie, personal fees and non‐financial support from AstraZeneca, personal fees and non‐financial support from Berlin‐Chemie, personal fees and non‐financial support from Chiesi, personal fees from EMMA, personal fees from GSK, personal fees from HAL Allergy, personal fees from Sunpharm, personal fees from HVD, personal fees from LEK‐AM, personal fees from Polpharma, personal fees from Teva, personal fees from Sanofi, personal fees from Zentiva, personal fees from Stada, outside the submitted work. JC. Ivancevich reports personal fees from Laboratorios Casasco Argentina, outside the submitted work. A. Todo‐Bom reports personal fees from GSK, grants and personal fees from LETI, personal fees from Mylan, grants from AbbVie, grants from Sanofi and grants from Roxal, outside the submitted work. L. Tuyet reports grants from AstraZeneca, grants from Boehringer Ingelheim, grants from Glaxo Smith Kline, grants from M.S.D., grants from Hyphens, grants from Tedis, grants from Novartis and grants from Orient Pharma, outside the submitted work. N. Papadopoulos reports grants from Nestle, Numil, Vianex, Vibrant, personal fees from Abbott, AstraZeneca, GSK, HAL, Menarini, Novartis, Danone Nutricia, OM Pharma, Regeneron, Sanofi, outside the submitted work. S. Toppila‐Salmi reports grants and other support from Sanofi Pharma, grants and other support from GSK, other support from OrionPharma, other support from ALK‐Abelló, other support from AstraZeneca, other support from Clario, outside the submitted work. R. Naclerio reports personal fees from Sanofi, personal fees from Lyra, outside the submitted work. M. Soyka reports other from Sanofi, other from M.S.D., other from GSK, other from AstraZeneca, outside the submitted work. N. Roche reports grants and personal fees from Boehringer Ingelheim, grants and personal fees from Novartis, grants and personal fees from GSK, personal fees from AstraZeneca, personal fees from Chiesi, grants and personal fees from Pfizer, personal fees from Sanofi, personal fees from Zambon, personal fees from M.S.D., personal fees from Austral, personal fees from Biosency, outside the submitted work. C. Suppli Ulrik reports personal fees from AZ, personal fees from GSK, grants and personal fees from BI, personal fees from TEVA, personal fees from Pfizer, personal fees from Orion, grants and personal fees from Sanofi, personal fees and non‐financial support from Novartis, personal fees from Chiesi, personal fees from Covis Pharma, personal fees from Berlin‐Chemie, outside the submitted work. D. Chu reports being a GRADE Working Group member, supports the development of allergy guidelines internationally and holds an E.J. Moran Campbell Career Award and a CIHR Inclusive Excellence Prize in Patient Engagement. Y. Okamoto reports personal fees from Torii Pharmaceutical Co. Ltd., personal fees from Tanabe Mitsubishi Pharmaceutical Co. Ltd., personal fees from Kirin Holdings Co. Ltd., personal fees from Shionogi Co. Ltd., personal fees from Stallergenes Greer and personal fees from Diichi‐Sankyo, outside the submitted work. H. Olze received fees (lectures, advisory boards, research grants) from F. Hoffmann‐La Roche Ltd., Sanofi‐Aventis Deutschland GmbH, AstraZeneca GmbH, GlaxoSmithKline GmbH and Co. KG and Novartis, outside the present work. J. Mullol reports personal fees and other from Sanofi‐Genzyme and Regeneron, grants, personal fees and other from Viatris/Meda Pharma, grants and personal fees from Noucor/Uriach Group, personal fees from Menarini, personal fees from UCB, personal fees and other from AstraZeneca, grants, personal fees and other from GSK, personal fees from M.S.D., personal fees and other from Lilly, personal fees and other from Glenmark, outside the submitted work. M. Torres reports personal fees from Leti Laboratories, personal fees from Aimmune Therapeutics, personal fees from Diater Laboratories, grants from European Commission, grants from SEAIC, grants from ISCIII, outside the submitted work. H. Kraxner reports Speaker's fee and congress support from Sanofi, Speaker's fee and congress support from Viatris, Speaker's fee and congress support from Berlin‐Chemie, Speaker's fee and congress support from Ewopharma; Advisory Board membership: Sanofi. M. Makris reports personal fees from ELPEN, personal fees from MENARINI, personal fees from AstraZeneca, personal fees from Novartis, personal fees from CHIESI, personal fees from GSK, personal fees from ThermoFisher, personal fees from Vianex, outside the submitted work. D. Sakurai reports grants and personal fees from Torii, grants and personal fees from Tanabe Mitsubishi, personal fees from Shionogi, grants and personal fees from Taiho, grants and personal fees from Kyorin, personal fees from Meiji Seika Pharma, personal fees from Thermo Fisher Scientific Diagnostics, grants and personal fees from Tsumura, personal fees from Hisamitsu, personal fees from Novartis, personal fees from Sanofi, personal fees from AstraZeneca, outside the submitted work. J. Correia‐de‐Sousa reports other from Boheringer Ingelheim, personal fees and other from GSK, grants, personal fees and other from AstraZeneca, personal fees from Bial, non‐financial support from Mundipharma, personal fees from Sanofi, other from Novartis, personal fees from M.S.D., personal fees from Medinfar, outside the submitted work. M. Bourgoin‐Heck reports non‐financial support from Thermofisher, personal fees from ALK, personal fees from Takeda, personal fees from Biocryst, personal fees from DBV, outside the submitted work. O. Palomares received research grants from MINECO, Ministerio de Ciencia e Innovación, CAM, Inmunotek S.L., Novartis and AstraZeneca and fees for giving scientific lectures or participation in Advisory Boards from AstraZeneca, Pfizer, GlaxoSmithKline, Inmunotek S.L., Novartis and Sanofi‐Genzyme. M. Moniuszko reports having received in the past personal fees and other support from Berlin‐Chemie/Menarini, personal fees and other support from AstraZeneca, personal fees and other support from GlaxoSmithKline, personal fees and other support from Novartis, personal fees and other support from Chiesi, personal fees and other support from Celon Pharma, personal fees and other support from Takeda, personal fees and other support from Polfarmex, personal fees and other support from CSL Behring, personal fees from Glenmark Pharmaceuticals, personal fees and other support from Sanofi, outside the submitted work. D. Larenas Linnemann reports personal fees from ALK, Armstrong, Astrazeneca national and global, Bayer, Chiesi, Grunenthal, Grin, GSK national and global, Viatris, Menarini, M.S.D., Novartis, Pfizer, Sanofi, Siegfried, Carnot, Syneos Health, grants from Abbvie, Bayer, Lilly, Sanofi, Astrazeneca, Pfizer, Novartis, Pulmonair, GSK, Chiesi, Biopharma, outside the submitted work; and Editor‐in‐Chief of Immune System (Karger)—member of asthma committee ACAAI—subgroup chair of allergen immunotherapy Practice Parameter Update JTF AAAAI/ACAAI 2024—member of allergen immunotherapy committee AAAAI—chair of allergen immunotherapy committee CMICA—member of allergic asthma task force EAACI. M. Worm reports other from AbbVie Deutschland GmbH & Co. KG, other from Aimmune Therapeutics UK Limited, other from ALK‐Abelló Arzneimittel GmbH, other from Allergopharma GmbH & Co KG, other from Almirall Hermal GmbH, other from Amgen GmbH, other from AstraZeneca GmbH, other from Bayer AG, other from Bencard Allergy GmbH, other from Bioprojet Pharma, other from Boehringer Ingelheim Pharma GmbH & Co. KG, other from Bristol Myers Squibb GmbH & Co. KGaA, other from Galderma Laboratorium GmbH, other from Glaxo Smith Kline GmbH & Co. KG, other from Infectopharm Arzneimittel und Consilium GmbH, other from LEO Pharma GmbH, other from Lilly Deutschland GmbH, other from Mylan Germany GmbH (A Viatris Company), other from Novartis AG, other from Octapharm AG, other from Pfizer Pharma GmbH, other from Sanofi‐Aventis Deutschland GmbH/Ðenzyme Europe B. B., outside the submitted work. T. Kosak Soklic reports personal fees from Meda AB, outside the submitted work. P. Devillier reports personal fees from ALK‐Abello, personal fees and non‐financial support from Stallergenes, personal fees and non‐financial support from AstraZeneca, personal fees from Chiesi, personal fees from GlaxoSmithKline, personal fees from Menarini, personal fees from Viatris, personal fees from Procter & Gamble Health, outside the submitted work. L. Cecchi reports personal fees from Menarini, personal fees from Chiesi, personal fees from AstraZeneca, personal fees from GSK, personal fees from Novartis, personal fees from Thermofisher, personal fees from Sanofi, outside the submitted work. G. Roberts reports grants from the National Institute of Health Research, grants from the National Institutes of Health, grants from Action Medical Research, grants from EU IMI2, other support from ALK‐Abello, non‐financial support and other support from ThermoFisher, other support from AstraZeneca, other support from Viatris, outside the submitted work. E. Compalati reports personal fees from Lofarma spa during the conduct of the study. D. Choudhury reports personal fees from Athaneum, others from ESPGHAN, others from the British Society of Allergy and Clinical Immunology (BSACI) ‐ Standards of Care Committee, others from Respiratory Allergy, International Primary Care Respiratory Group, outside the submitted work. R. Buhl reports personal fees from AstraZeneca, Berlin‐Chemie, Celltrion, Chiesi, Cipla, Sanofi and Teva, as well as grants to Mainz University Hospital and personal fees from Boehringer Ingelheim, GlaxoSmithKline, Novartis and Roche, outside the submitted work. L. de las Vecillas reports fees for Lectures/Advisory Boards from AstraZeneca, GSK, LETI labs and Allergy Therapeutics, outside the submitted work. She has received a Research grant from the Spanish Society of Allergy and Clinical Immunology Foundation and from the ISCIII, outside the submitted work. L. Taborda‐Barata reports personal fees from LETI, personal fees from Sanofi and personal fees from Diater, outside the submitted work. J. Bernstein reports personal fees from Pfizer, personal fees from Lilly and personal fees from Sanofi. S. Del Giacco reports grants and personal fees from AstraZeneca, grants and personal fees from GSK, grants and personal fees from Sanofi, personal fees from Chiesi, personal fees from Menarini, outside the submitted work. M. Giovannini reports personal fees from Sanofi, from Thermo Fisher Scientific, outside the submitted work. N. Miculinic reports personal fees from Salvus d.o.o. Pharma Industry, personal fees from Viatris Ltd., outside the submitted work. J. da Silva reports grants, personal fees and others from Novartis, grants and personal fees from Takeda, outside the submitted work. G. Paoletti reports other from LoFarma, other from GSK and other from Astrazeneca, outside the submitted work. H. Schünemann reports and develops guidelines on Allergic Rhinitis in Asthma (ARIA), and his academic institution received research funding for it. The other authors declare no conflicts of interest, outside the submitted work.

## Supporting information


**Appendix S1:** all70100‐sup‐0001‐AppendixS1.docx.

## Data Availability

The data that support the findings of this study are available from the corresponding author upon reasonable request.
