# Supplementary material for: Methodology for the Development of the Allergic Rhinitis and Its Impact on Asthma (ARIA)‐EAACI 2024–2025 Guidelines: From Evidence‐to‐Decision Frameworks to Digitalised Shared Decision‐Making Algorithms
Source: Allergy. 2025 Nov 21;81(2):427–53. doi: 10.1111/all.70100 (PMC12862529; doi:10.1111/all.70100)
Supplement: Supplementary file 1 — Appendix S1: all70100‐sup‐0001‐AppendixS1.docx. [file ALL-81-427-s001.docx]

**ONLINE SUPPLEMENT**

**Supplementary Table 1. Findings from MASK-air^®^ studies that were relevant for the ARIA 2024-2025 guidelines**

| **1. Behaviour of patients with allergic rhinitis**  i. When the full database is analysed:   - Most patients are not adherent to treatment^1^ and rather use as-needed treatment^2,3^ (i.e., do not take medication when they are feeling well-controlled^2,3^), - Most patients increase the number of medications based on symptoms. This has been observed in different European countries ^2^ ^4^ and with treatments bought in the pharmacies, ^2,5^ - Patients use co-medication when they are feeling less well-controlled (as reported in cross-sectional and longitudinal analyses). ^2,6-9^ - Patient’s satisfaction to treatment is lower in co-medication than in monotherapy. ^10,11^   ii. When only patients adherent to the app are analysed:   - Patients are often adherent to medications; adherence to the app appears to be associated with adherence to medications. - Except for intranasal corticosteroids, increased adherence is not associated with an increased level of control. - Switching of medications is common, as displayed in longitudinal analyses. ^12^   **2. Patients’ values and preferences**   - Poor rhinitis control is associated with lower quality-of-life, most notably with lower utilities and values of the EQ-5D visual analogue scale.^13^   **3. Resources required**   - Poor rhinitis control is associated with higher work impairment, resulting in indirect costs (due to productivity losses) that can reach hundreds of US dollars per week.^14^   **4. Acceptability**   - In adjusted analyses, and when used in monotherapy, intranasal corticosteroids and fixed combinations of intranasal antihistamines + corticosteroids were associated with increased satisfaction compared to intranasal antihistamines and oral antihistamines (work in preparation). - In adjusted analyses, intranasal antihistamines were associated with higher odds of being used in co-medication when compared to intranasal corticosteroids, fixed combinations of intranasal antihistamines + corticosteroids, and oral antihistamines. In addition, intranasal corticosteroids also tended to be more frequently used in co-medication than fixed combinations of intranasal antihistamines + corticosteroids or oral antihistamines (work in preparation). - In complete weeks of MASK-air reporting during the pollen season, there were 36.0% in which intranasal corticosteroids were used for 6 or 7 days. This compares with 38.5% for oral antihistamines and 31.7% for fixed combinations of intranasal antihistamines + corticosteroids (work in preparation). |
| --- |

**Supplementary Table 2. ARIA 2024-2025 group members and their tasks**

| 1. **Steering Committee**   The Steering Committee is formed by:   - Jean Bousquet - Holger Schünemann - Bernardo Sousa Pinto - Boleslaw Samolinski - Alkis Togias - Torsten Zuberbier  1. **Guideline Panel**   The Guideline Panel is formed by:   - Ana Luísa Neves - Ana Margarida Pereira - Anna Bedbrook - Antonio Bognanni - Arunas Valiulis - Bernardo Sousa-Pinto - Cristina Jacomelli - Elena Azzolini - Elena Parmelli - G. Walter Canonica - Gilles Louis - Holger Schünemann - Jaron Zuberbier - Jean Bousquet - Juan Jose Yepes Nuñez - Leticia de las Vecillas - Lucas Leemann - Ludger Klimek - Maria Jose Torres - Maria Teresa Ventura - Marine Savoure - Mark Dykewicz - Martin Hoffman-Apitius - Nikolaos Papadopoulos - Olga Lourenço - Rafael José Vieira - Sanna Toppila Salmi - Sian Williams - Torsten Zuberbier - Yuliia Palamarchuk   The tasks of the Guideline Panel are:   - To propose, review and prioritise PICO questions; - To propose, review and prioritise outcomes; - To propose judgements for the different criteria of the Evidence-to-Decision framework; - To formulate guideline recommendations; - To participate in the conception of the digitalised management algorithms; - To disseminate ARIA in their country.   At least 50% of the members of the Guideline panel do not have any conflicts of interest according to the Guidelines International Network Conflicts of Interest declaration and management principles reviewed by *Arbeitsgemeinschaft der Wissenschaftlichen Medizinischen Fachgesellschaften e. V.*   1. **Review Group**   This larger group includes ARIA members and Presidents (or delegates) of scientific societies that will endorse ARIA 2024-2025.  Its tasks are:   - To review the list of potentially prioritised and non-prioritised questions and to assess whether they have any concerns related to the specific questions which have or have not been prioritised. - To present specificities of their countries in terms of management, healthcare system as well as medication availability and affordability. A special effort will be made for low- and middle-income countries. - To review the formulation of the recommendations. - To translate the ARIA 2024-2025 executive summary. - To disseminate ARIA in their countries.  1. **ARIA Junior Members (below 35 years of age)**   There is an urgent need to involve young people and increase diversity in ARIA.  The tasks of the ARIA junior members are:   - To review the formulation of the recommendations. - To translate the ARIA 2024-2025 executive summary. - To disseminate ARIA in their countries. |
| --- |

**Supplementary Table 3. Stepwise processes for understanding the differences between rhinitis alone *versus* rhinitis + asthma** (expanded from ^15^)

1. A clinical observation led to the concept that multimorbidity (allergic rhinitis [AR] + asthma) and IgE polysensitisation may be linked. ^16^
2. Mechanistic ^17^ and epidemiologic studies (European Community Respiratory Health Survey: ECRHS, Framework Programme , FP2) ^18^ allowed us to better understand the links between asthma and AR. The concept of “one-airway-one-disease” ^19^ led to the ARIA guidelines. ^20^
3. AR alone affects around 70-80% of AR patients whereas AR + asthma multimorbidity affects 20-30%. ^21^ On the other hand, most asthma patients have allergic or non-allergic rhinitis. ^22-24^ These data suggested common pathways between AR+asthma and rhinitis-specific pathways.
4. The severity of AR and asthma increases in parallel on exposure to an allergen. ^24^
5. The EU network of excellence GA^2^LEN (Global Allergy and Asthma European Network, FP6) ^25^ attempted to better understand sensitisation patterns. ^26^
6. MeDALL (Mechanisms of the Development of Allergy, FP7) ^27,28^ led to considerable improvement in understanding the mechanisms underlying the complex interactions between multimorbidity and polysensitisation (epidemiologic, genomic and epigenetic studies). ^26^
7. Overall, AR+asthma is associated with polysensitisation in Europe, ^29-39^, New Zealand, ^40^ Brazil ^41^ and China. ^42,43^
8. Moreover, by comparison to mono-sensitised patients, polysensitised ones have an earlier age of onset, more commonly an uncontrolled disease, higher eosinophil counts and total IgE levels ^44-46^ as well as different trajectories. ^30,47-49^
9. Impact of AR on asthma: Concomitant AR in asthma patients appears to contribute to increased asthma exacerbations and poorer asthma control, both in children ^50-54^ and adults globally. ^55-66^
10. MASK-air® (Mobile Airways Sentinel networK), an mHealth (mobile health) app, helped to capture direct patient-reported data to obtain novel insights into the complex interactions informed by MeDALL. ^67^
11. Clustering MASK-air® data using AR or asthma identified an extreme allergy phenotype including AR + asthma + conjunctivitis. This phenotype was more severe than single diseases alone. However, mHealth observational studies are only hypothesis generating. ^68^
12. Canonical epidemiologic studies showed that (i) the risk of adult-onset asthma increases with the number of allergic comorbidities, ^38^ (ii) severe asthma is associated with multimorbidity, ^69^ (iii) age of onset and parental allergy suggest that multimorbidity behaves differently to AR or asthma alone, ^44,45^ and (iv) the role of sex hormones at puberty appears to be influenced by multimorbidity.^70,71^
13. A discovery study in MeDALL (Europe, transcriptomics and RT-PCR) and a validation study in EVA-PR (Puerto-Rico, RNA sequencing) yielded the same results: Multimorbidity (asthma, AR, atopic dermatitis) was associated with 7 genes of T2 signalling: *IL-5* (eosinophils) and *IL-33* (polysensitisation and eosinophilia). ^72^ In MeDALL, AR-specific genes have been identified. These genes are mostly associated with TLR signalling pathways and *IL-17*. ^72,73^ There is almost no overlap between AR alone and AR+ asthma. ^73^ In children and adolescents, no specific genes could be identified in asthma or atopic dermatitis alone. ^72^
14. There are shared epigenetic patterns of allergic multimorbidities but, in the MeDALL study, in children, these epigenetic patterns were not found in asthma alone. ^74-76^ In nasal brushed cells in childhood, strong DNA-methylation signatures were shared by the AR + asthma phenotype, ^76^ confirming previous findings in blood.
15. An extreme allergy phenotype (AR + asthma + conjunctivitis or AR + asthma + atopic dermatitis) is rare ^77-80^ but occurs more commonly than by chance. This extreme phenotype was confirmed by MASK-air® and associated with lack of control and severity. ^12,81,82^
16. The new ARIA-MeDALL hypothesis was confirmed in a case control and an epidemiologic study in the general population (Constances). In Constances, participants with AR + asthma had more severe AR symptoms than those with AR alone^83,84^ as well as an earlier age of onset^84,85^. These results were replicated in the case control study EGEA (epidemiological study on the genetics and environment of asthma, bronchial hyperresponsiveness and atopy). ^86^
17. There are therapeutic consequences related to this hypothesis. In Constances, for the four ARIA classes, participants with AR + asthma more often reported a treatment with intranasal corticosteroids and oral antihistamines than those with AR alone.^87^ In MASK-air^®^, a co-medication pattern was associated with a poorer AR control than monotherapy, ^9^ suggesting that AR+asthma participants of the Constances cohort were less well controlled than those with AR alone. ^7,88^
18. MASK-air^®^ direct patient data support the ARIA-MeDALL hypothesis on allergic phenotypes that (i) were found to be similar in 13 different countries in Europe, Turkey, Brazil and Mexico, ^89^ (ii) were confirmed in AR ^12^ or asthma ^82^ clustering, and (iii) had an impact on work productivity, ^81,90^ EQ-5D utility index score or EQ-5D VAS. ^14^

**Supplementary Box 1. Criteria that we asked panel members to consider when rating the priority of questions**

| We asked panel members to consider a question being of higher priority if it was one:   - That commonly arises in practice; - For which there is uncertainty in practice regarding how to manage patients; - For which there is new research evidence to consider; - That is associated with variation in practice; - That has important consequences for, or is associated with, high resource use or costs; - That has not been previously or sufficiently addressed (e.g., in previous guidelines). |
| --- |

**Supplementary Box 2. Example of health outcome descriptor (outcome of nasal symptoms)**

| Nasal symptoms Population: Intermittent Allergic Rhinitis  **Symptoms**  You experience nasal symptoms. These symptoms may include runny nose, which is when your nose is running with a clear, watery discharge. You may also have nasal congestion, which is when your nose feels stuffy and blocked. You could also have nasal itching, which may make you want to rub or scratch your nose. Sneezing is also a common symptom, and you may find yourself sneezing more often than usual. The more severe your symptoms, the more you will be bothered by them.  **Time Horizon**  Your symptoms can come and go. You will experience nasal symptoms less than 4 days a week or less than 4 weeks in a row.  **Testing and Treatment**  You can often manage your nasal symptoms with over-the-counter or prescription medications. These medications may be taken intranasally or orally. If your symptoms are severe or impair your daily activities, a healthcare provider may recommend immunotherapy which can be given under your tongue or by injection to help reduce your allergic reactions over time. Nasal symptoms may often be assessed by using patient diaries which reflect the past 12 or 24 hours.  **Consequences**  Nasal symptoms may be bothersome and affect your daily life. They may lead to sleep disturbances, decreased productivity, and reduced overall quality of life. However, with proper management and treatment, your nasal symptoms can often be controlled, allowing you to enjoy a better quality of life and relief from the discomfort they cause.  Population: Persistent Allergic Rhinitis  **Symptoms**  You experience nasal symptoms. These symptoms may include runny nose, which is when your nose is running with a clear, watery discharge. You may also have nasal congestion, which is when your nose feels stuffy and blocked. You could also have nasal itching, which may make you want to rub or scratch your nose. Sneezing is also a common symptom, and you may find yourself sneezing more often than usual. The more severe your symptoms, the more you will be bothered by them.  **Time Horizon**  You will experience nasal symptoms at least 4 days a week and 4 weeks in a row.  **Testing and Treatment**  You can often manage your nasal symptoms with over-the-counter or prescription medications. These medications may be taken intranasally or orally. If your symptoms are severe or impair your daily activities, a healthcare provider may recommend immunotherapy which can be given under your tongue or by injection to help reduce your allergic reactions over time. Nasal symptoms may often be assessed by using patient diaries which reflect the past 12 or 24 hours.  **Consequences**  Nasal symptoms may be bothersome and affect your daily life. They may lead to sleep disturbances, decreased productivity, and reduced overall quality of life. However, with proper management and treatment, your nasal symptoms can often be controlled, allowing you to enjoy a better quality of life and relief from the discomfort they cause. |
| --- |

**Supplementary Figure 1. Example of treatment algorithms of previous ARIA guidelines**

**
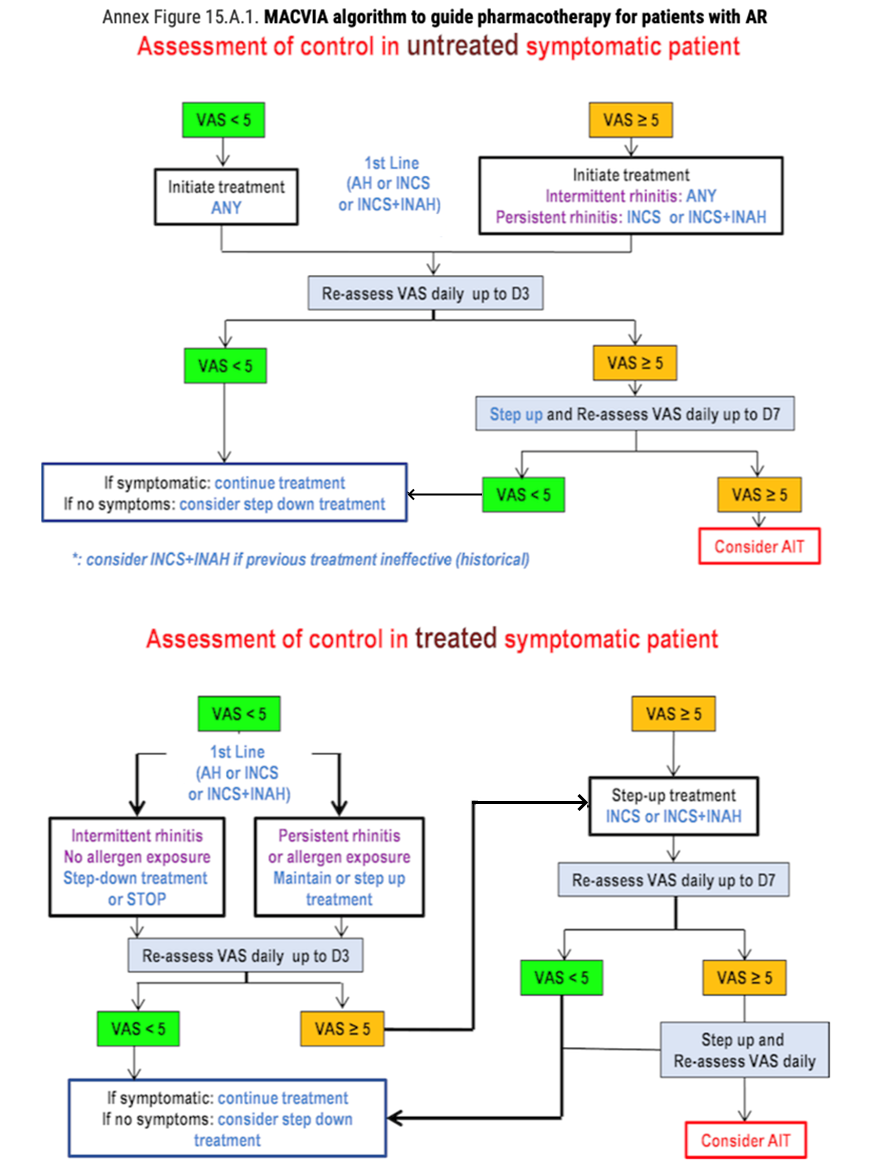
**

AH=Antihistamines; AIT=Allergen immunotherapy; D=Day; INAH=Intranasal antihistamines; INCS=Intranasal corticosteroids; VAS=Visual analogue scale

**References**

- 1. Menditto E, Costa E, Midao L, et al. Adherence to treatment in allergic rhinitis using mobile technology. The MASK Study. *Clin Exp Allergy*. Apr 2019;49(4):442–460. doi:10.1111/cea.13333
- 2. Sousa-Pinto B, Sa-Sousa A, Vieira RJ, et al. Behavioural patterns in allergic rhinitis medication in Europe: A study using MASK-air((R)) real-world data. *Allergy*. Mar 8 2022;77(9):2699–2711. doi:10.1111/all.15275
- 3. Bousquet J, Klimek L, Kuna P, Mullol J, Toppila-Salmi S. The Debate: Regular Versus As-Needed Use of Intranasal Corticosteroids for a Patient-Centered Approach. *J Allergy Clin Immunol Pract*. Mar 2021;9(3):1374–1375. doi:10.1016/j.jaip.2020.11.019
- 4. Bousquet J, Agache I, Anto JM, et al. Google Trends terms reporting rhinitis and related topics differ in European countries. *Allergy*. Aug 2017;72(8):1261–1266. doi:10.1111/all.13137
- 5. Bousquet J, Schroder-Bernhardi D, Bachert C, et al. Heterogeneity of the pharmacologic treatment of allergic rhinitis in Europe based on MIDAS and OTCims platforms. *Clin Exp Allergy*. Aug 2021;51(8):1033–1045. doi:10.1111/cea.13884
- 6. Bousquet J, Devillier P, Arnavielhe S, et al. Treatment of allergic rhinitis using mobile technology with real-world data: The MASK observational pilot study. *Allergy*. Sep 2018;73(9):1763–1774. doi:10.1111/all.13406
- 7. Bedard A, Basagana X, Anto JM, et al. Mobile technology offers novel insights into the control and treatment of allergic rhinitis: The MASK study. *J Allergy Clin Immunol*. Jul 2019;144(1):135–143 e6. doi:10.1016/j.jaci.2019.01.053
- 8. Bedard A, Basagana X, Anto JM, et al. Treatment of allergic rhinitis during and outside the pollen season using mobile technology. A MASK study. *Clin Transl Allergy*. Dec 9 2020;10(1):62. doi:10.1186/s13601-020-00342-x
- 9. Sousa-Pinto B, Schunemann HJ, Sa-Sousa A, et al. Comparison of rhinitis treatments using MASK-air(R) data and considering the Minimal Important Difference. *Allergy*. May 14 2022;77(10):3002–3014. doi:10.1111/all.15371
- 10. Sousa-Pinto B, Vieira RJ, Bognanni A, et al. Comparison of Allergic Rhinitis Treatments on Patient Satisfaction: A MASK-air and EAACI Methodological Committee Report. *Allergy*. Sep 26 2025;doi:10.1111/all.70055
- 11. Vieira RJ, Sousa-Pinto B, Gil-Mata S, et al. Comparing allergic rhinitis treatments based on patient satisfaction: A MASK-air(R) study. *J Allergy Clin Immunol Pract*. Jun 6 2025;doi:10.1016/j.jaip.2025.05.058
- 12. Sousa-Pinto B, Schunemann HJ, Sa-Sousa A, et al. Consistent trajectories of rhinitis control and treatment in 16,177 weeks: The MASK-air(R) longitudinal study. *Allergy*. Apr 2023;78(4):968–983. doi:10.1111/all.15574
- 13. Vieira RJ, Leemann L, Briggs A, et al. Poor rhinitis and asthma control is associated with decreased health-related quality-of-life and utilities: A MASK-air study. *J Allergy Clin Immunol Pract*. Mar 30 2024;doi:10.1016/j.jaip.2024.03.036
- 14. Vieira RJ, Azevedo LF, Pereira AM, et al. Impact of allergic rhinitis control on work productivity and costs: a real-world data MASK-air study. *J Allergy Clin Immunol Pract*. Aug 5 2024;doi:10.1016/j.jaip.2024.07.026
- 15. Bousquet J, Melen E, Haahtela T, et al. Rhinitis associated with asthma is distinct from rhinitis alone: The ARIA-MeDALL hypothesis. *Allergy*. May 2023;78(5):1169–1203. doi:10.1111/all.15679
- 16. Bousquet J, Coulomb Y, Arrendal H, Robinet-Levy M, Michel FB. Total serum IgE concentrations in adolescents and adults using the phadebas IgE PRIST technique. *Allergy*. 1982;37(6):397–406.
- 17. Vignola AM, Chanez P, Godard P, Bousquet J. Relationships between rhinitis and asthma. *Allergy*. 1998;53(9):833–9.
- 18. Leynaert B, Bousquet J, Neukirch C, Liard R, Neukirch F. Perennial rhinitis: An independent risk factor for asthma in nonatopic subjects: Results from the European Community Respiratory Health Survey. *J Allergy Clin Immunol*. 1999:301–304.
- 19. Simons FE. Allergic rhinobronchitis: the asthma-allergic rhinitis link. *J Allergy Clin Immunol*. 1999;104(3 Pt 1):534–40.
- 20. Bousquet J, Van Cauwenberge P, Khaltaev N. Allergic rhinitis and its impact on asthma. *J Allergy Clin Immunol*. 2001;108(5 Suppl):S147–334.
- 21. Leynaert B, Neukirch C, Kony S, et al. Association between asthma and rhinitis according to atopic sensitization in a population-based study. *J Allergy Clin Immunol*. Jan 2004;113(1):86–93.
- 22. Togias A, Gergen PJ, Hu JW, et al. Rhinitis in children and adolescents with asthma: Ubiquitous, difficult to control, and associated with asthma outcomes. *J Allergy Clin Immunol*. Mar 2019;143(3):1003–1011 e10. doi:10.1016/j.jaci.2018.07.041
- 23. Cruz AA, Popov T, Pawankar R, et al. Common characteristics of upper and lower airways in rhinitis and asthma: ARIA update, in collaboration with GA(2)LEN. *Allergy*. 2007;62 Suppl 84:1–41. doi:ALL1551 [pii]
- 10.1111/j.1398-9995.2007.01551.x
- 24. Togias AG. Systemic immunologic and inflammatory aspects of allergic rhinitis. *J Allergy Clin Immunol*. Nov 2000;106(5 Suppl):S247–50.
- 25. Bousquet J, Burney PG, Zuberbier T, et al. GA2LEN (Global Allergy and Asthma European Network) addresses the allergy and asthma 'epidemic'. *Allergy*. Jul 2009;64(7):969–77. doi:10.1111/j.1398-9995.2009.02059.x
- 26. Bousquet J, Anto JM, Bachert C, et al. Factors responsible for differences between asymptomatic subjects and patients presenting an IgE sensitization to allergens. A GALEN project. *Allergy*. Jun 2006;61(6):671–80.
- 27. Bousquet J, Anto J, Auffray C, et al. MeDALL (Mechanisms of the Development of ALLergy): an integrated approach from phenotypes to systems medicine. *Allergy*. May 2011;66(5):596–604. doi:10.1111/j.1398-9995.2010.02534.x
- 28. Anto JM, Bousquet J, Akdis M, et al. Mechanisms of the Development of Allergy (MeDALL): Introducing novel concepts in allergy phenotypes. *J Allergy Clin Immunol*. Feb 2017;139(2):388–399. doi:10.1016/j.jaci.2016.12.940
- 29. Westman M, Lupinek C, Bousquet J, et al. Early childhood IgE reactivity to pathogenesis-related class 10 proteins predicts allergic rhinitis in adolescence. *J Allergy Clin Immunol*. May 2015;135(5):1199–206 e1–11. doi:10.1016/j.jaci.2014.10.042
- 30. Asarnoj A, Hamsten C, Waden K, et al. Sensitization to cat and dog allergen molecules in childhood and prediction of symptoms of cat and dog allergy in adolescence: A BAMSE/MeDALL study. *J Allergy Clin Immunol*. Mar 2016;137(3):813–21 e7. doi:10.1016/j.jaci.2015.09.052
- 31. Mikkelsen S, Dinh KM, Boldsen JK, et al. Combinations of self-reported rhinitis, conjunctivitis, and asthma predicts IgE sensitization in more than 25,000 Danes. *Clin Transl Allergy*. Mar 2021;11(1):e12013. doi:10.1002/clt2.12013
- 32. Filiou A, Holmdahl I, Asarnoj A, et al. Development of Sensitization to Multiple Allergen Molecules from Preschool to School Age Is Related to Asthma. *Int Arch Allergy Immunol*. Jan 18 2022:1–12. doi:10.1159/000521324
- 33. Blondal V, Malinovschi A, Sundbom F, et al. Multimorbidity in asthma, association with allergy, inflammatory markers and symptom burden, results from the Swedish GA(2) LEN study. *Clin Exp Allergy*. Feb 2021;51(2):262–272. doi:10.1111/cea.13759
- 34. Schoos AM, Jelding-Dannemand E, Stokholm J, Bonnelykke K, Bisgaard H, Chawes BL. Single and multiple time-point allergic sensitization during childhood and risk of asthma by age 13. *Pediatr Allergy Immunol*. Nov 2019;30(7):716–723. doi:10.1111/pai.13109
- 35. Raciborski F, Bousquet J, Bousqet J, et al. Dissociating polysensitization and multimorbidity in children and adults from a Polish general population cohort. *Clin Transl Allergy*. 2019;9:4. doi:10.1186/s13601-019-0246-y
- 36. Schmidt F, Hose AJ, Mueller-Rompa S, et al. Development of atopic sensitization in Finnish and Estonian children: A latent class analysis in a multicenter cohort. *J Allergy Clin Immunol*. May 2019;143(5):1904–1913 e9. doi:10.1016/j.jaci.2018.12.1014
- 37. Hose AJ, Depner M, Illi S, et al. Latent class analysis reveals clinically relevant atopy phenotypes in 2 birth cohorts. *J Allergy Clin Immunol*. Jun 2017;139(6):1935–1945 e12. doi:10.1016/j.jaci.2016.08.046
- 38. Toppila-Salmi S, Chanoine S, Karjalainen J, Pekkanen J, Bousquet J, Siroux V. Risk of adult-onset asthma increases with the number of allergic multimorbidities and decreases with age. *Allergy*. Dec 2019;74(12):2406–2416. doi:10.1111/all.13971
- 39. Bengtsson C, Lindberg E, Jonsson L, et al. Chronic Rhinosinusitis Impairs Sleep Quality: Results of the GA2LEN Study. *Sleep*. Jan 1 2017;40(1)doi:10.1093/sleep/zsw021
- 40. Sears MR, Burrows B, Flannery EM, Herbison GP, Holdaway MD. Atopy in childhood. I. Gender and allergen related risks for development of hay fever and asthma. *Clin Exp Allergy*. Nov 1993;23(11):941–8.
- 41. Aranda CS, Cocco RR, Pierotti FF, et al. Allergic sensitization pattern of patients in Brazil. *J Pediatr (Rio J)*. Jul–Aug 2021;97(4):387–395. doi:10.1016/j.jped.2020.08.005
- 42. Zhang W, Xie B, Liu M, Wang Y. Associations between sensitisation to allergens and allergic diseases: a hospital-based case-control study in China. *BMJ open*. Feb 1 2022;12(2):e050047. doi:10.1136/bmjopen-2021-050047
- 43. Gao Z, Fu WY, Sun Y, et al. Artemisia pollen allergy in China: Component-resolved diagnosis reveals allergic asthma patients have significant multiple allergen sensitization. *Allergy*. Feb 2019;74(2):284–293. doi:10.1111/all.13597
- 44. Burte E, Bousquet J, Siroux V, Just J, Jacquemin B, Nadif R. The sensitization pattern differs according to rhinitis and asthma multimorbidity in adults: the EGEA study. *Clin Exp Allergy*. Feb 25 2017;doi:10.1111/cea.12897
- 45. Siroux V, Ballardini N, Soler M, et al. The asthma-rhinitis multimorbidity is associated with IgE polysensitization in adolescents and adults. *Allergy*. Jul 2018;73(7):1447–1458. doi:10.1111/all.13410
- 46. Kauffmann F, Dizier MH, Annesi-Maesano I, et al. EGEA (Epidemiological study on the Genetics and Environment of Asthma, bronchial hyperresponsiveness and atopy)-- descriptive characteristics. *Clin Exp Allergy*. Dec 1999;29 Suppl 4:17–21.
- 47. Asarnoj A, Hamsten C, Lupinek C, et al. Prediction of peanut allergy in adolescence by early childhood storage protein-specific IgE signatures: The BAMSE population-based birth cohort. *J Allergy Clin Immunol*. Feb 09 2017;doi:10.1016/j.jaci.2016.12.973
- 48. Ballardini N, Bergstrom A, Wahlgren CF, et al. IgE antibodies in relation to prevalence and multimorbidity of eczema, asthma, and rhinitis from birth to adolescence. *Allergy*. Mar 2016;71(3):342–9. doi:10.1111/all.12798
- 49. Wickman M, Lupinek C, Andersson N, et al. Detection of IgE Reactivity to a Handful of Allergen Molecules in Early Childhood Predicts Respiratory Allergy in Adolescence. *EBioMedicine*. Dec 2017;26:91–99. doi:10.1016/j.ebiom.2017.11.009
- 50. Kang HY, Park CS, Bang HR, Sazonov V, Kim CJ. Effect of allergic rhinitis on the use and cost of health services by children with asthma. *Yonsei Med J*. Aug 30 2008;49(4):521–9. doi:10.3349/ymj.2008.49.4.521
- 51. de Groot EP, Nijkamp A, Duiverman EJ, Brand PL. Allergic rhinitis is associated with poor asthma control in children with asthma. *Thorax*. Jul 2012;67(7):582–7. doi:10.1136/thoraxjnl-2011-201168
- 52. Padilla J, Uceda M, Ziegler O, Lindo F, Herrera-Perez E, Huicho L. Association between allergic rhinitis and asthma control in Peruvian school children: a cross-sectional study. *Biomed Res Int*. 2013;2013:861213. doi:10.1155/2013/861213
- 53. Pongracic JA, Krouse RZ, Babineau DC, et al. Distinguishing characteristics of difficult-to-control asthma in inner-city children and adolescents. *J Allergy Clin Immunol*. Oct 2016;138(4):1030–1041. doi:10.1016/j.jaci.2016.06.059
- 54. Butz AM, Bellin M, Tsoukleris M, et al. Very Poorly Controlled Asthma in Urban Minority Children: Lessons Learned. *J Allergy Clin Immunol Pract*. May–Jun 2018;6(3):844–852. doi:10.1016/j.jaip.2017.08.007
- 55. Halpern MT, Schmier JK, Richner R, Guo C, Togias A. Allergic rhinitis: a potential cause of increased asthma medication use, costs, and morbidity. *J Asthma*. Feb 2004;41(1):117–26.
- 56. Bousquet J, Gaugris S, Kocevar VS, et al. Increased risk of asthma attacks and emergency visits among asthma patients with allergic rhinitis: a subgroup analysis of the improving asthma control trial. *Clin Exp Allergy*. Jun 2005;35(6):723–7.
- 57. Gaugris S, Sazonov-Kocevar V, Thomas M. Burden of concomitant allergic rhinitis in adults with asthma. *J Asthma*. Feb 2006;43(1):1–7.
- 58. Ponte EV, Franco R, Nascimento HF, et al. Lack of control of severe asthma is associated with co-existence of moderate-to-severe rhinitis. *Allergy*. May 2008;63(5):564–9. doi:10.1111/j.1398-9995.2007.01624.x
- 59. Magnan A, Meunier JP, Saugnac C, Gasteau J, Neukirch F. Frequency and impact of allergic rhinitis in asthma patients in everyday general medical practice: a French observational cross-sectional study. *Allergy*. Mar 2008;63(3):292–8. doi:10.1111/j.1398-9995.2007.01584.x
- 60. Vandenplas O, Dramaix M, Joos G, et al. The impact of concomitant rhinitis on asthma-related quality of life and asthma control. *Allergy*. Oct 2010;65(10):1290–7. doi:10.1111/j.1398-9995.2010.02365.x
- 61. Ohta K, Bousquet PJ, Aizawa H, et al. Prevalence and impact of rhinitis in asthma. SACRA, a cross-sectional nation-wide study in Japan. Research Support, Non-U.S. Gov't. *Allergy*. Oct 2011;66(10):1287–95. doi:10.1111/j.1398-9995.2011.02676.x
- 62. Bin Mahfouz T, Banjar SA, Assiri RA, Alshehri GA, Binyousef F. The Prevalence and Impact of Allergic Rhinitis on Asthma Exacerbations in Asthmatic Adult Patients in the Riyadh Region of Saudi Arabia: A Cross-Sectional Study. *Cureus*. Dec 2022;14(12):e32324. doi:10.7759/cureus.32324
- 63. Megersa S, Chala G, Fikremariam K. Determinants of Asthma Attack Among Adult Asthmatic Patients Attending at Public Hospitals of West Shoa Zone, Oromia Regional State, Ethiopia, 2021: Case-Control Study. *J Asthma Allergy*. 2022;15:1143–1154. doi:10.2147/JAA.S376499
- 64. Geleta LA, Dadi LS, Sona AA. Determinants of uncontrolled asthma among adult asthmatic patients on follow-up at chest clinic of Jimma medical center: unmatched case-control study. *J Asthma*. Jun 2022;59(6):1103–1109. doi:10.1080/02770903.2021.1908351
- 65. Sriprasart T, Saiphoklang N, Kawamatawong T, et al. Allergic rhinitis and other comorbidities associated with asthma control in Thailand. *Front Med (Lausanne)*. 2023;10:1308390. doi:10.3389/fmed.2023.1308390
- 66. Scelo G, Torres-Duque CA, Maspero J, et al. Analysis of comorbidities and multimorbidity in adult patients in the International Severe Asthma Registry. *Ann Allergy Asthma Immunol*. Jan 2024;132(1):42–53. doi:10.1016/j.anai.2023.08.021
- 67. Bousquet J, Bedbrook A, Czarlewski W, et al. Guidance to 2018 good practice: ARIA digitally-enabled, integrated, person-centred care for rhinitis and asthma. *Clin Transl Allergy*. 2019;9:16. doi:10.1186/s13601-019-0252-0
- 68. Bousquet J, Anto JM, Bachert C, et al. ARIA digital anamorphosis: Digital transformation of health and care in airway diseases from research to practice. *Allergy*. Jan 2021;76(1):168–190. doi:10.1111/all.14422
- 69. Toppila-Salmi S, Lemmetyinen R, Chanoine S, et al. Risk factors for severe adult-onset asthma: a multi-factor approach. *BMC Pulm Med*. Jul 8 2021;21(1):214. doi:10.1186/s12890-021-01578-4
- 70. Frohlich M, Pinart M, Keller T, et al. Is there a sex-shift in prevalence of allergic rhinitis and comorbid asthma from childhood to adulthood? A meta-analysis. *Clin Transl Allergy*. 2017;7:44. doi:10.1186/s13601-017-0176-5
- 71. Rosario CS, Cardozo CA, Neto HJC, Filho NAR. Do gender and puberty influence allergic diseases? *Allergol Immunopathol (Madr)*. 2021;49(2):122–125. doi:10.15586/aei.v49i2.49
- 72. Lemonnier N, Melen E, Jiang Y, et al. A novel whole blood gene expression signature for asthma, dermatitis, and rhinitis multimorbidity in children and adolescents. *Allergy*. Apr 11 2020;75:3248–60. doi:10.1111/all.14314
- 73. Aguilar D, Lemonnier N, Melen E, et al. Distinction between rhinitis alone and rhinitis with asthma using interactomics. *Sci Rep*. Aug 12 2023;13(1):13125. doi:10.1038/s41598-023-39987-6
- 74. Xu CJ, Soderhall C, Bustamante M, et al. DNA methylation in childhood asthma: an epigenome-wide meta-analysis. *Lancet Respir Med*. May 2018;6(5):379–388. doi:10.1016/S2213-2600(18)30052-3
- 75. Xu CJ, Gruzieva O, Qi C, et al. Shared DNA methylation signatures in childhood allergy: The MeDALL study. *J Allergy Clin Immunol*. Mar 2021;147(3):1031–1040. doi:10.1016/j.jaci.2020.11.044
- 76. Qi C, Jiang Y, Yang IV, et al. Nasal DNA methylation profiling of asthma and rhinitis. *J Allergy Clin Immunol*. Jun 2020;145(6):1655–1663. doi:10.1016/j.jaci.2019.12.911
- 77. Pinart M, Benet M, Annesi-Maesano I, et al. Comorbidity of eczema, rhinitis, and asthma in IgE-sensitised and non-IgE-sensitised children in MeDALL: a population-based cohort study. *Lancet Respir Med*. Feb 2014;2(2):131–40. doi:10.1016/S2213-2600(13)70277-7
- 78. Garcia-Aymerich J, Benet M, Saeys Y, et al. Phenotyping asthma, rhinitis and eczema in MeDALL population-based birth cohorts: an allergic comorbidity cluster. *Allergy*. Aug 2015;70(8):973–84. doi:10.1111/all.12640
- 79. Pols DHJ, Nielen MMJ, Korevaar JC, Bindels PJE, Bohnen AM. Reliably estimating prevalences of atopic children: an epidemiological study in an extensive and representative primary care database. *NPJ Prim Care Respir Med*. Apr 13 2017;27(1):23. doi:10.1038/s41533-017-0025-y
- 80. Pols DH, Wartna JB, van Alphen EI, et al. Interrelationships between Atopic Disorders in Children: A Meta-Analysis Based on ISAAC Questionnaires. *PloS one*. 2015;10(7):e0131869. doi:10.1371/journal.pone.0131869
- 81. Bousquet J, Devillier P, Anto JM, et al. Daily allergic multimorbidity in rhinitis using mobile technology: A novel concept of the MASK study. *Allergy*. Aug 2018;73(8):1622–1631. doi:10.1111/all.13448
- 82. Bousquet J, Sousa-Pinto B, Anto JM, et al. Identification by cluster analysis of patients with asthma and nasal symptoms using the MASK-air(R) mHealth app. *Pulmonology*. Nov 22 2022;doi:10.1016/j.pulmoe.2022.10.005
- 83. Savoure M, Bousquet J, Leynaert B, et al. Rhinitis phenotypes and multimorbidities in the general population: the CONSTANCES cohort. *Eur Respir J*. Feb 2023;61(2)doi:10.1183/13993003.00943-2022
- 84. Savoure M, Bousquet J, Leynaert B, et al. Rhinitis phenotypes and multimorbidities in the general population Constances cohort. *Eur Respir J*. Oct 6 2022;doi:10.1183/13993003.00943-2022
- 85. Burte E, Bousquet J, Siroux V, Just J, Jacquemin B, Nadif R. The sensitization pattern differs according to rhinitis and asthma multimorbidity in adults: the EGEA study. *Clin Exp Allergy*. Feb 25 2017;47(4):520–529. doi:10.1111/cea.12897
- 86. Burte E, Bousquet J, Varraso R, et al. Characterization of Rhinitis According to the Asthma Status in Adults Using an Unsupervised Approach in the EGEA Study. *PloS one*. 2015;10(8):e0136191. doi:10.1371/journal.pone.0136191
- 87. Savoure M, Bousquet J, Leynaert B, et al. Asthma is associated with increased severity and duration of rhinitis: A study with the Allergic Rhinitis and its Impact on Asthma classes in the Constances cohort. *Clin Transl Allergy*. Nov 2023;13(11):e12316. doi:10.1002/clt2.12316
- 88. Sousa-Pinto B, Sa-Sousa A, Vieira RJ, et al. Behavioural patterns in allergic rhinitis medication in Europe: A study using MASK-air((R)) real-world data. *Allergy*. Mar 8 2022;doi:10.1111/all.15275
- 89. Bousquet J, Sousa-Pinto B, Regateiro FS, et al. MASK-air(R) direct patient data support the ARIA-MeDALL hypothesis on allergic phenotypes. *Allergy*. Aug 11 2023;doi:10.1111/all.15842
- 90. van de Schoot R, Depaoli S, King R, et al. Bayesian statistics and modelling. *Nature Reviews Methods Primers*. 2021;1(1):1.
